# Intracranial venous thrombosis imaging by anatomical location: an educational review with mimics and diagnostic pitfalls

**DOI:** 10.1186/s13244-026-02381-7

**Published:** 2026-09-18

**Authors:** Anna Falk Delgado, David Fällmar, Heather Martin, Michael V. Mazya, Fabian Arnberg, Annika Kits

**Affiliations:** 1 Karolinska Institutet, Department of Clinical Neuroscience, Stockholm, Sweden; 2 Karolinska University Hospital, Department of Neuroradiology, Stockholm, Sweden; 3 Uppsala University, Department of Surgical Sciences, Neuroradiology, Uppsala, Sweden; 4 Karolinska University Hospital, Department of Neurology, Stockholm, Sweden

**Keywords:** Intracranial venous thrombosis, Cerebral venous sinus thrombosis, Dural sinus thrombosis, Venous lake thrombosis, Neuroimaging

## Abstract

**Abstract:**

Intracranial venous thrombosis encompasses diverse etiologies with clinical presentations ranging from an incidental finding to coma and death. Despite potentially devastating consequences, venous thrombosis is frequently overlooked by radiologists due to its predominantly extraaxial location close to the skull and clinical emphasis on arterial imaging evaluation in acute stroke management. This educational review addresses common and uncommon presentations across all anatomical locations of intracranial venous thrombosis, providing practical pearls and pitfalls for diagnostic interpretation. We review the imaging modalities CT and MRI, together with their venographic techniques, correlating imaging findings with anatomical location, thrombus age and signal evolution, complications, and underlying causes. Critical interpretive pitfalls are highlighted with strategies to avoid misdiagnosis. The review also emphasizes the important yet underrecognized association between intracranial pressure disorders and venous thrombosis, providing radiologists with a practical framework for improved diagnostic accuracy.

**Key Points:**

- **Question** How can radiologists systematically recognize intracranial venous thrombosis across all anatomical locations and avoid the diagnostic pitfalls that lead to clinically significant missed diagnoses?
- **Findings** Location-specific imaging patterns, thrombus signal evolution, and recognition of common mimics such as arachnoid granulations enable accurate detection using combined non-contrast and venographic sequences.
- **Critical relevance statement** By organizing intracranial venous thrombosis imaging anatomically and pairing each location with its specific mimics and pitfalls, this review equips radiologists with a practical framework to improve detection accuracy and prevent clinically significant missed diagnoses.

**Graphical Abstract:**

**Figure:**
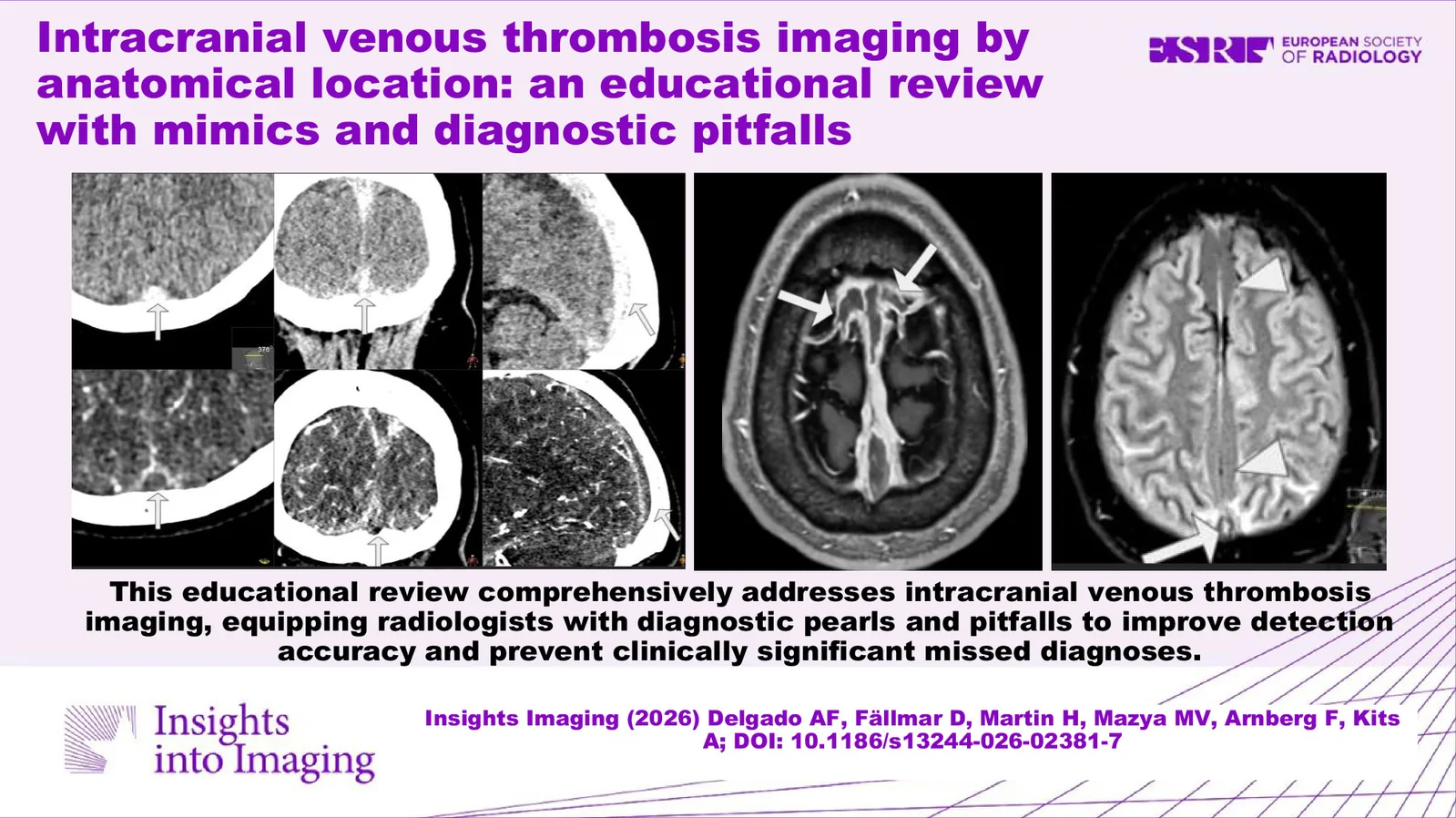

## Introduction

Cerebral venous thrombosis (CVT) is the formation of a thrombus in any intracranial venous structure, accounting for approximately 0.5%–3% of all strokes [1, 2]. It differs fundamentally from arterial stroke in its diverse etiologies and clinical presentation. Unlike arterial stroke, symptomatic venous thrombosis typically presents with a gradual onset of symptoms, reflecting the time required for venous pressure elevation to alter cerebral physiology and drainage patterns. Where arterial ischemia produces near-instantaneous symptoms with loss of function, venous thrombosis more frequently causes headaches, seizures, and increased intracranial pressure [3].

While cerebral veins have collateral anastomotic drainage routes similar to arterial collaterals, this protective mechanism is less developed in cortical veins than in dural sinuses. Consequently, cortical vein thrombosis more frequently causes symptomatic venous stasis than thrombosis of larger dural sinuses [4]. Ultimately, severe venous drainage impairment can produce brain tissue damage comparable to arterial ischemia, albeit within a more protracted time course.

Despite the multifactorial nature of CVT, the concept of Virchow’s triad (endothelial injury, stasis and hypercoagulability) still explains many cases [5]. Risk factors for CVT include both inherited and acquired conditions (Table 1) [1, 6–9]. CVT affects individuals of any age, with a mean age around 30–45 years and a female predominance [1, 2]. The prognosis has improved with enhanced detection and treatment strategies, with up to 80%–90% remaining functionally independent, 57% experiencing no residual symptoms, and 8–15% suffering severe disability or death [1, 7, 10].

**Table 1:** Risk factors and predisposing conditions for intracranial venous thrombosis

Inherited thrombophilia	Acquired prothrombotic states	Hormonal and hematological	Mechanical and infectious	Systemic conditions
Factor V Leiden mutation	Antiphospholipid syndrome	Pregnancy and puerperium	Skull fracture/head trauma	Dehydration
Prothrombin G20210A mutation	Myeloproliferative disorders	Oral contraceptives	Neurosurgery/cerebral venous catheterization	Sepsis
Protein C or S deficiency	Malignancy (hematological malignancies, solid tumors)	Hormone replacement therapy	Otitis media/mastoiditis/sinusitis /facial infection	Inflammatory bowel disease
Antithrombin III deficiency	Nephrotic syndrome	Polycythemia	Meningitis/encephalitis	Systemic lupus erythematosus
	Medications (L-asparaginase, steroids, androgens)	Severe anemia	Intracranial hypotension	Behçet’s disease
	Paroxysmal nocturnal hemoglobinuria		Tumor compression or overgrowth	Sarcoid
	Vaccine-induced immune thrombocytopenia			Ulcerative colitis
				Rheumatological conditions

Despite being clinically feared and radiologically overlooked, CVT is increasingly recognizable using systematic imaging interpretation, improved imaging protocols, and awareness of diagnostic pitfalls [11, 12]. This educational review illustrates venous thrombosis across various intracranial locations, highlights key imaging pearls and pitfalls, and provides practical guidance on optimal imaging strategies, including principles of thrombus dating and signal evolution.

## Dating of intracranial venous thrombosis by imaging technique

The imaging characteristics of intracranial venous thrombus follow general principles of signal evolution that parallel those of parenchymal hematoma [13], particularly in the acute and subacute stages, as both primarily reflect hemoglobin degradation within red blood cells. A venous thrombus consists primarily of red blood cells entrapped in a fibrin network [14]. As plasma is expelled during clot retraction, the resulting cellular concentration increases attenuation on computed tomography (CT) and alters magnetic resonance imaging (MRI) signal characteristics.

On non-enhanced CT, acute thrombus typically appears hyperdense, reflecting the high concentration of hemoglobin within densely packed red blood cells. As the thrombus organizes, the densely packed red blood cells are progressively replaced by collagen, fibrin, and hemosiderin deposits, reducing cellular density and causing CT attenuation to fall toward, and eventually below, that of flowing blood [15, 16].

On MRI, signal evolution is governed by hemoglobin degradation and can be broadly divided into three stages, though the boundaries between stages are approximate and vary considerably among patients and even within thrombosed segments of the same individual [17, 18]. In the acute stage, intracellular deoxyhemoglobin renders the thrombus isointense on T1-weighted and hypointense on T2-weighted imaging. This appearance closely mimics the normal venous flow void on conventional sequences and represents a significant source of diagnostic error if standard T1 and T2 sequences are interpreted in isolation. However, three-dimensional T1-weighted black-blood imaging overcomes this limitation by suppressing intravascular flow signal, improving thrombus conspicuity even in the acute stage [19]. SWI and GRE sequences are also sensitive at this stage, demonstrating marked hypointensity and blooming due to the paramagnetic effects of deoxyhemoglobin. As deoxyhemoglobin is oxidized to methemoglobin in the subacute stage, the T1 signal increases markedly. In the early subacute phase, this is accompanied by T2 hypointensity, reflecting intracellular methemoglobin, while in the late subacute phase, extracellular methemoglobin results in hyperintensity on both T1 and T2-weighted sequences [18]. In the chronic stage, signal intensity returns toward isointensity on T1 and becomes iso to hyperintense on T2, reflecting progressive organization of the thrombus and its replacement by vascularized connective tissue [20]. Partial or complete recanalization may occur, restoring venous flow, though some degree of chronic luminal change may persist [21]. The imaging characteristics of each modality, including recommended protocols, diagnostic pearls and pitfalls, are summarized in Table 2.

**Table 2:** Imaging protocol, pearls, and pitfalls in intracranial venous thrombosis

CT	Pearl	Pitfall	Strategy
Non-contrast	A focal hyperattenuating sinus with focal sinus expansion has high specificity for venous thrombosis. Focal low HU in the sinus can indicate old thrombus	General hyperattenuating venous sinuses can have physiological or external causes (for example, previous contrast agent injection). Lower sensitivity in the sigmoid sinus due to streak artifacts from bone. Misses subacute and chronic thrombus	Correlate with venography. Measure HU values in neonates and compare with the rest of the venous system. Search for previous contrast agent injection. Cannot confidently exclude a venous thrombus.
CTV	Contrast enhancement, delineating a thrombus, has high specificity	Early venous phase, or asymmetric venous filling, may mimic thrombosis. Interpreting CTV without native information may cause false positives in the arachnoid granulation of the hypoplastic sinus. The cavernous sinus fills later than other dural sinuses (45 s delay). Requires contrast administration	Use adequate venous delay (20–25 s standard, 45 s for cavernous sinus); compare with non-enhanced CT. Consider multiphase venography if unsure. Considered comparable to contrast-enhanced MRV for CVST.
Multiphase CT-venography	Aids perfect timing in one of the phases	Radiation exposure. Requires contrast administration	Multiphase imaging reduces timing-related misinterpretations if imaged from the arterial to the late venous phase. Concern with radiation dose
**MRI**	**Pearl**	**Pitfall**	**Strategy**
T1 (preferably black blood)	Subacute thrombus has a high signal on T1	Hyperacute thrombus, before a significant increase in T1 signal, is harder to detect. High T1-signal in hemorrhage	Signal evolution varies and is affected by the flow phenomenon. Look for loss of flow hyperechoic signal in acute thrombus. High signal in subacute thrombus up to one month.
T1+gd	May fill the patent lumen and leave the thrombus with relatively low signaling	Contrast media may obscure the thrombus in the late-contrast phase. Chronic thrombus with delayed enhancement may mimic a patent sinus on parenchymal phase images. Requires contrast administration	Cannot exclude a venous thrombus.
T2/T2FLAIR	Shows edema and parenchymal complications; the thrombus signal varies with evolution, but is generally a high signal	Cannot reliably distinguish vasogenic from cytotoxic edema alone	Combine with DWI for edema characterization
SWI/GRE	Excellent for cortical vein thrombosis and venous stasis (dilated medullary veins)	Fails in the late subacute phase (extracellular methemoglobin); proximity to bone obscures high convexity veins	Recommended for cortical vein thrombosis. Sensitive for acute-early subacute cortical vein thrombosis
DWI	Restricted diffusion in thrombus confirms acute thrombosis; it differentiates cytotoxic from vasogenic edema	Not all thrombi show restricted diffusion	Differentiate cytotoxic from vasogenic edema, can visualize a fresh thrombus with a high signal
TOF-MRV or Phase imaging	Non-contrast technique; useful in pregnancy	Flow gaps are common in hypoplastic sinuses and arachnoid granulations; slow flow mimics thrombosis; subacute thrombus T1 shine-through	Flow-dependent, often does not give more information than high-resolution T2 images; sensitive to slow flow artifacts. Limited specificity
Contrast-MRV	Superior to TOF; better for cortical vein thrombosis	Filling defect on venography in the arachnoid granulation, hypoplastic sinus and early venous timing. Chronic thrombus with delayed enhancement may mimic a patent sinus on parenchymal-phase images. Requires contrast administration	Superior to TOF for assessing thrombus vs slow flow. Recommended for cortical vein thrombosis. Concern with gadolinium in pregnant women. Valuable for detecting narrowing of the lateral venous sinuses in IIH. Use time-resolved imaging when available
Time-resolved contrast-enhanced MR-venography	Demonstrates sequential venous filling; eliminates timing artifacts	Requires contrast administration	Optimal for timing-related pitfalls if imaged from the arterial to the late venous phase. Concern with gadolinium in pregnant women. Valuable for detecting narrowing of the lateral venous sinuses in IIH or arachnoid granulation
High-resolution 3D T2-weighted sequences (CISS/FIESTA)	Detects subtle IIH signs (perioptic CSF distension, empty sella, flattened globes)	Not specific for thrombosis	Useful to assess for signs of increased intracranial pressure, such as flattened posterior orbital globes, papilledema, perioptic CSF distension, shallow pituitary gland in sella turcica (“empty sella”), and narrowing of the lateral venous sinuses

## Imaging of intracranial venous thrombosis by location

### Cerebral venous sinus thrombosis (CVST)

Dural sinuses are venous channels between the two dural layers, located primarily adjacent to the skull and draining mainly into the internal jugular veins at the skull base. The straight sinus is unique in not being in contact with the skull bone. CVST most commonly affects the transverse sinus, followed by the superior sagittal sinus (SSS) [7].

**Fig. 1:**
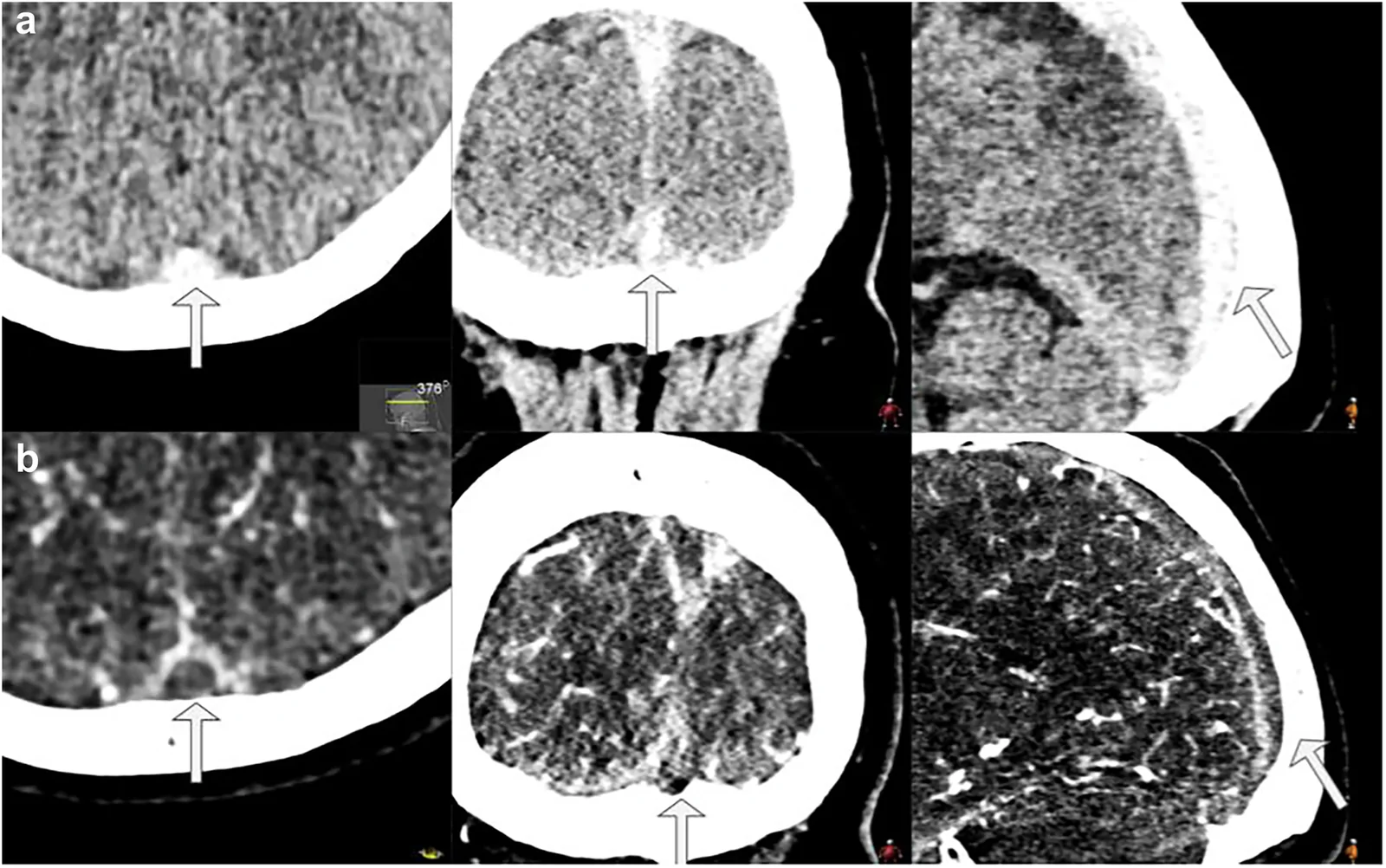
Acute superior sagittal sinus (SSS) thrombosis. **a** Non-enhanced CT shows hyperattenuation within the SSS (arrow), indicating acute thrombus. **b** CT venography demonstrates a filling defect in the SSS (arrow) with the absence of contrast opacification, confirming venous occlusion and resembling an “empty delta” sign

Acute thrombus typically fills the sinus lumen with high-attenuation on CT and iso to hyper T1-signal on MRI or a lack of normal venous flow voids (Fig. 1) [20, 22, 23]. Most MR images in any weighting are affected by a thrombus, varying with timing from onset due to the degradation of the thrombus [22, 23]. The computed tomography venography (CTV)/magnetic resonance venography (MRV) “empty delta” sign in the SSS entails venous enhancement around the thrombus [22], but can be false negative in cases where the intensity of the thrombus matches the surrounding contrast-enhancing agent (Sup Fig. 1).

**Fig. 2:**
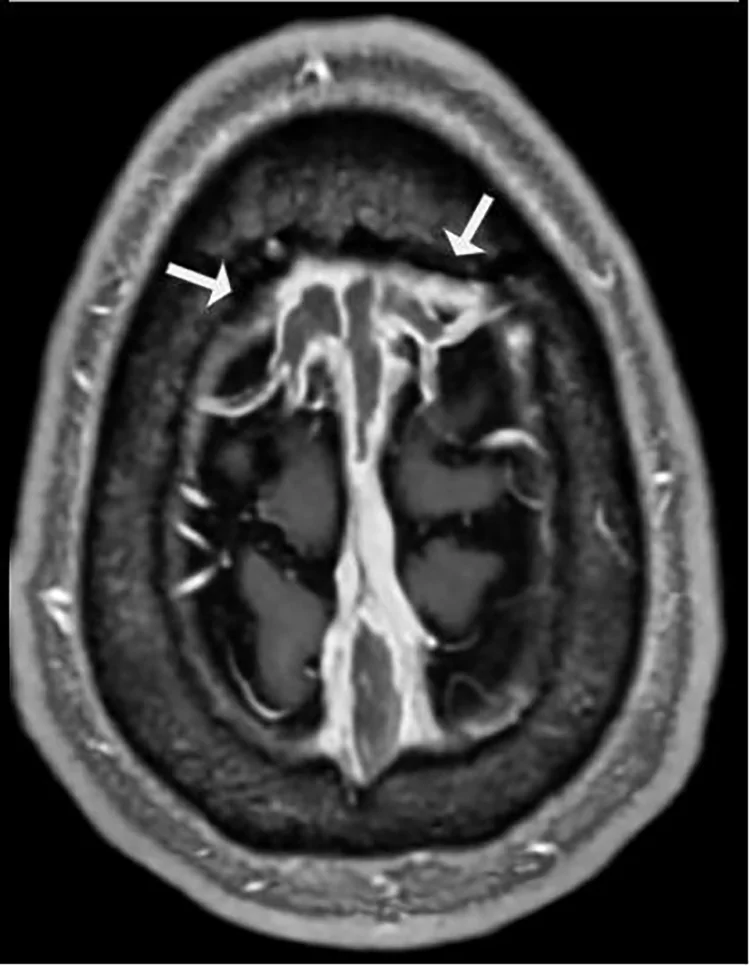
Contrast-enhanced T1-weighted MRI demonstrates filling defects delineating thrombus in the superior sagittal sinus extending into the bilateral bridging veins (arrows)

Venography demonstrates discontinuous contrast filling of the sinus at the site of the thrombus. The subsequent occlusion/stenosis causes venous stasis with slightly dilated, corkscrew-appearing medullary veins in the white matter (Sup Fig. 2). CVST may coexist with bridging- or cortical vein thrombosis (Fig. 2).

**Fig. 3:**
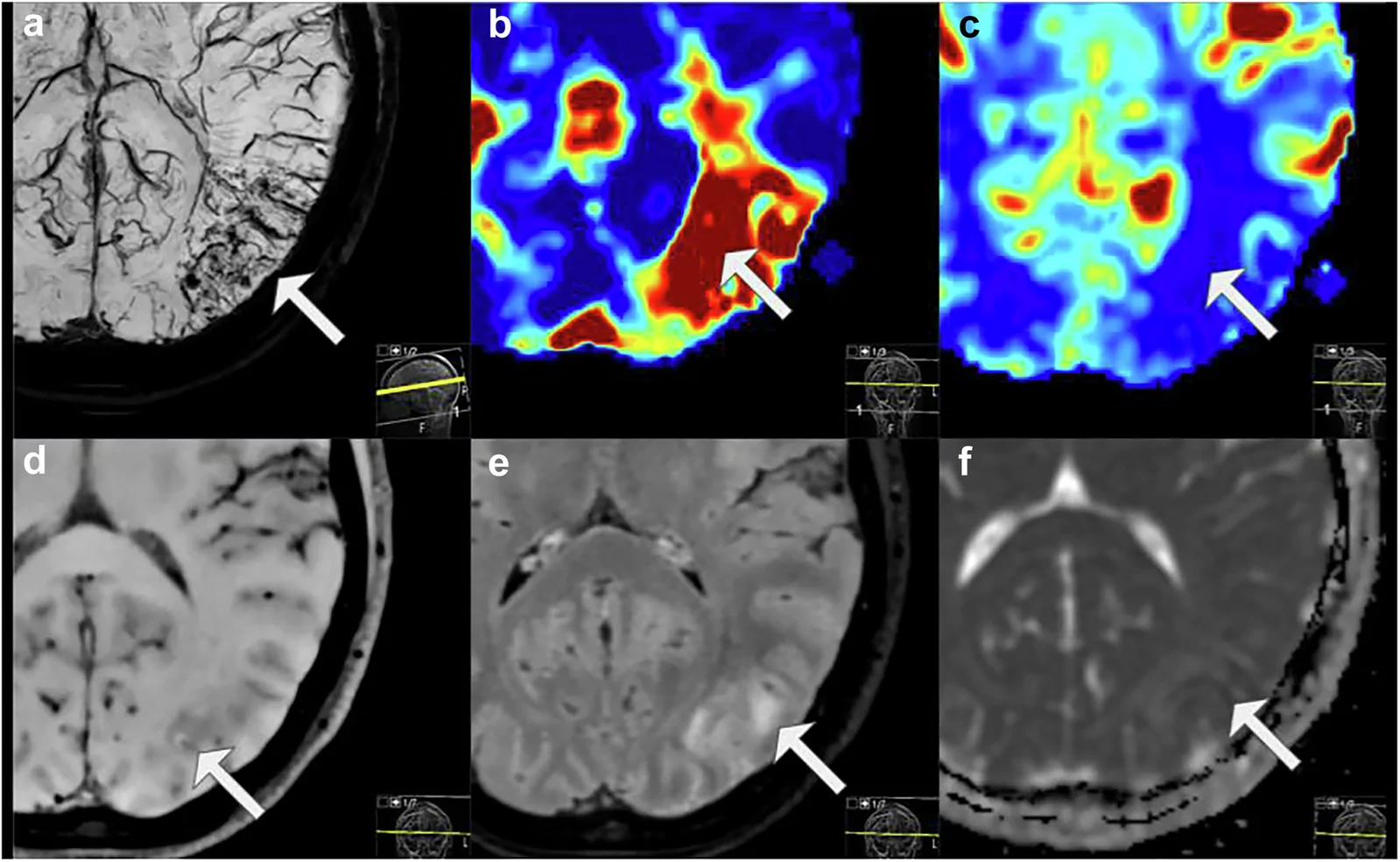
Extensive SSS and left transverse sinus thrombosis with multiple complications. **a** SWI shows corkscrew-dilated medullary veins (arrows) and microhemorrhages. **b**, **c** Perfusion imaging demonstrates prolonged MTT and reduced CBF in the left hemisphere. **d** Post-contrast T1 CUBE FS reveals subtle cortical hemorrhage (arrow). **e** T2-FLAIR shows vasogenic edema. **f** ADC map demonstrates focal cortical restricted diffusion (arrows), indicating mixed vasogenic and cytotoxic edema

**Fig. 4:**
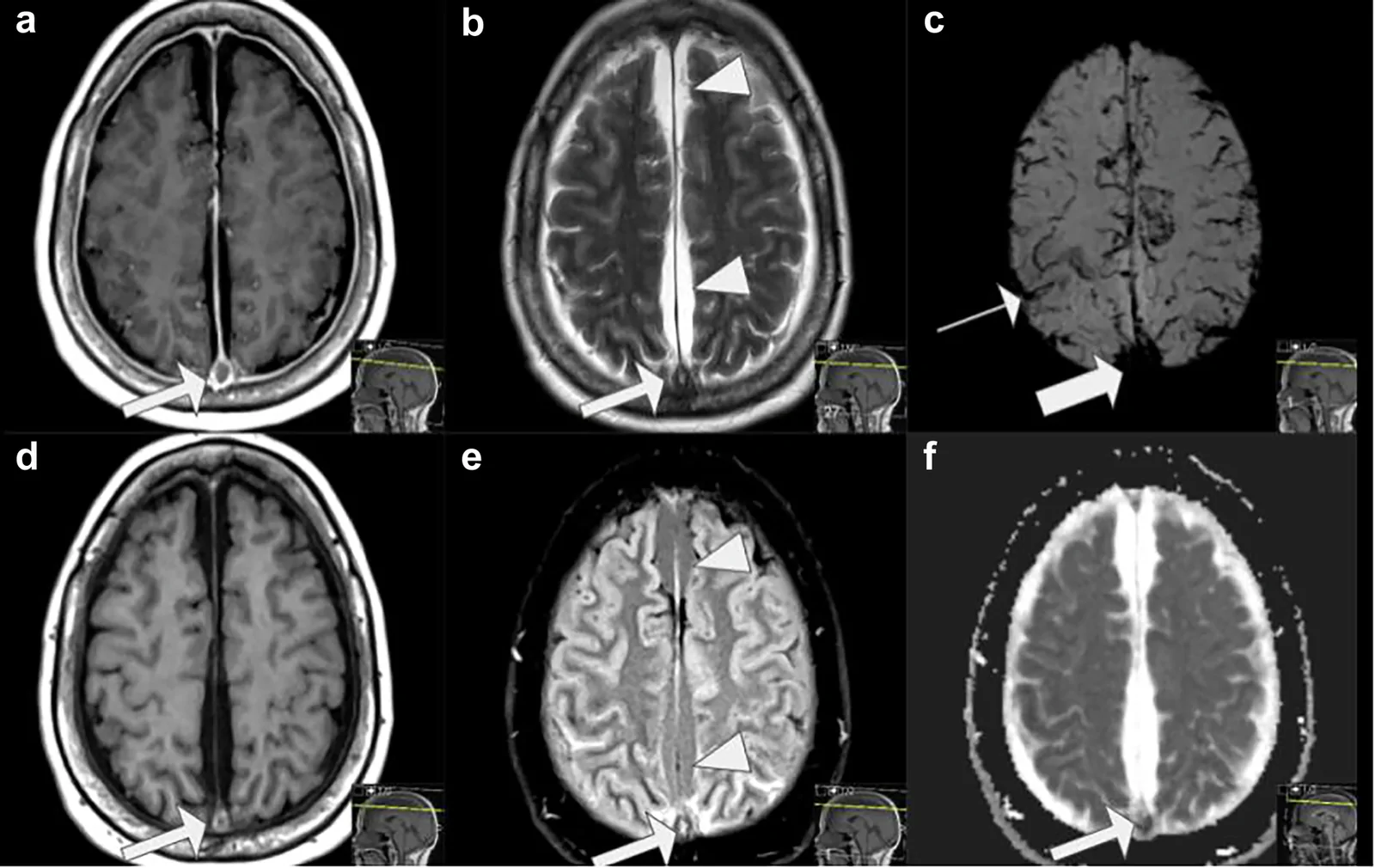
SSS thrombosis with subdural hygromas indicating intracranial hypotension. Multisequence MRI demonstrates: **a** Post-contrast T1 shows a filling defect with an empty delta sign (arrow). **b** T2-weighted image reveals heterogeneous signal in thrombus (arrow) and bilateral subdural hygromas with slightly elevated signal (arrowheads), not indicating frank hemorrhage. **c** SWI demonstrates dilated, stagnant cortical veins (thin arrows) and hypointense thrombus (thick arrow). **d** Non-enhanced T1 shows a heterogeneous signal within the thrombus (arrow). **e** T2-FLAIR demonstrates mixed signal intensity thrombus (arrow) and subdural hygromas with slight hyperintensity (arrowheads). **f** ADC map shows restricted diffusion within the SSS thrombus (arrow). The presence of subdural hygromas with venous stasis suggests underlying intracranial hypotension as a predisposing factor for thrombosis

Cerebral complications include vasogenic edema from increased transmural hydrostatic pressure, cytotoxic or intramyelin edema from reduced hypoxic perfusion pressure, and hemorrhage from microvascular rupture, possibly due to a combination of basal lamina disruption and increased hydrostatic pressure (Fig.  3) [24, 25]. Elevated intracranial pressure can cause papilledema and perioptic CSF distension (Sup Fig. 3). The straight sinus drains the deep venous system, including the internal cerebral veins, basal veins of Rosenthal, and vein of Galen. The absence of significant collateral drainage makes thrombosis of the straight sinus particularly devastating, causing deep hemispheric and thalamic edema, venous infarction, and hemorrhage, frequently resulting in altered mental status or coma (Sup Fig. 4 and Fig. 4).

#### Chronic cerebral venous sinus thrombosis

Chronic thrombosis presents differently, as collateral drainage compensates for occlusion. The thrombus loses its acute hyperdense CT or T1-hyperintense MRI appearance and instead shows heterogeneous attenuation on CT and variable signal intensity on MRI, with partial recanalization allowing some venous flow through the affected region (Sup Fig.  5) [20, 23]. Chronic thrombosis can also present on imaging as an abnormal appearance of blood. As seen in the example in (Sup Fig. 6).

### Secondary CVST from Adjacent or other Pathology

Otitis media with mastoiditis may be complicated by transosseous spread of infection to the adjacent sigmoid sinus (Sup Fig. 7). The infection can affect the venous sinus through three mechanisms: direct thrombosis induction, intraluminal filling with purulent material, or external compression by epidural abscess.

Skull fractures overlying the venous sinuses represent another common mechanism of thrombosis (Sup Fig. 8). Distinguishing intradural thrombus from an expanding epidural hematoma can be challenging [26].

**Fig. 5:**
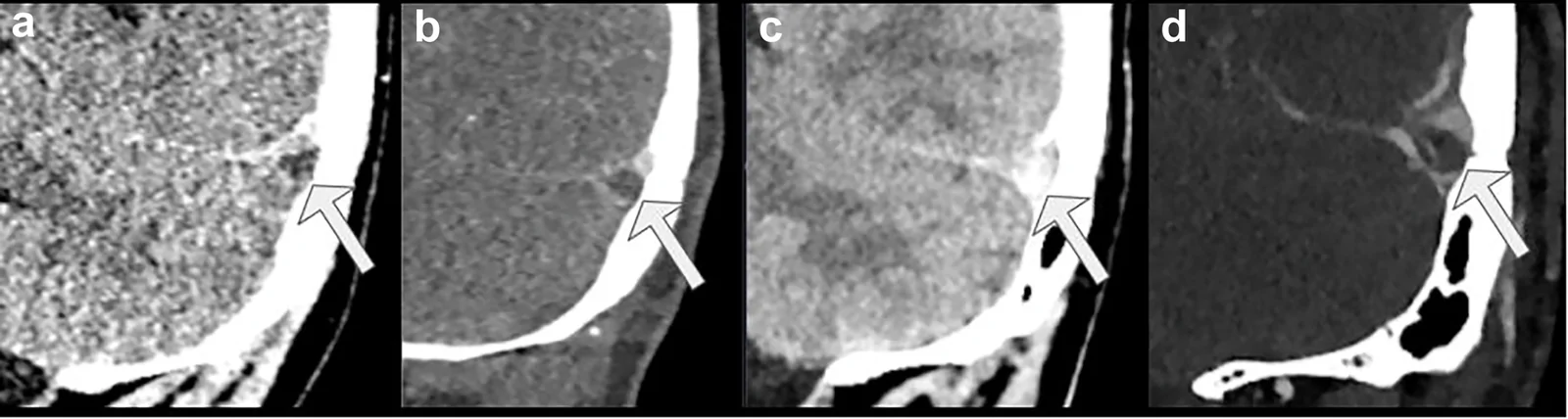
Evolution of blood-filled arachnoid granulation after subdural hemorrhage. **a** Non-enhanced CT shows normal CSF-attenuation arachnoid granulation in the left transverse sinus (arrow). **b** CT venography demonstrates a filling defect at the granulation site (arrow) with a tentorial subdural hematoma. **c** Follow-up non-enhanced CT shows hyperattenuating blood within the granulation (arrow). **d** Follow-up CT venography reveals a small vessel within the blood-filled granulation (arrow)

Intracranial hypotension has been independently associated with CVST [27] as seen in (Fig. 5).

#### Mimics and diagnostic pitfalls of CVST

Subdural hematoma delineating the dural layer with high-attenuating blood should not be confused with CVST (Sup Fig. 9).

Arachnoid granulations at typical locations, such as the vein of Labbé-transverse sinus junction, appear more rounded than the typical cord-like appearance of CVST and follow CSF signal characteristics (Sup Fig. 10) unless filled with subdural blood or containing herniated meninges, vessels, or brain parenchyma (Fig. 5).

Normal anatomical variants complicate interpretation, including a split superior sagittal sinus (SSS) (Sup Fig 11), inferior sagittal sinus aplasia, unilateral transverse sinus hypoplasia with asymmetric drainage patterns or occipital sinus. The torcular Herophili may lack tributaries from all adjacent sinuses, with straight or SSS drainage directed to only one transverse sinus. Further, the dural sinuses often have intrinsic septa, channeling the blood in separate compartments.

For dural tumors, specifically convexity meningiomas arising near the SSS, the venous sinus is often invaded or compressed by the growing tumor (Sup Fig. 12).

In neonates, venous sinuses commonly appear hyperdense for two reasons [28]. First, physiological polycythemia increases blood attenuation (Sup Fig 13). Second, the relatively high water content of the neonatal brain parenchyma makes the sinuses appear hyperdense by comparison. Thrombosed sinuses show higher attenuation than unaffected sinuses, whereas physiological causes affect all sinuses equally. In complex cases, Doppler ultrasound can be valuable in neonates [28]. In a pediatric population, Hounsfield unit measurements in non-thrombosed venous sinuses typically remain below 58, distinguishing them from thrombosis [29].

**Fig. 6:**
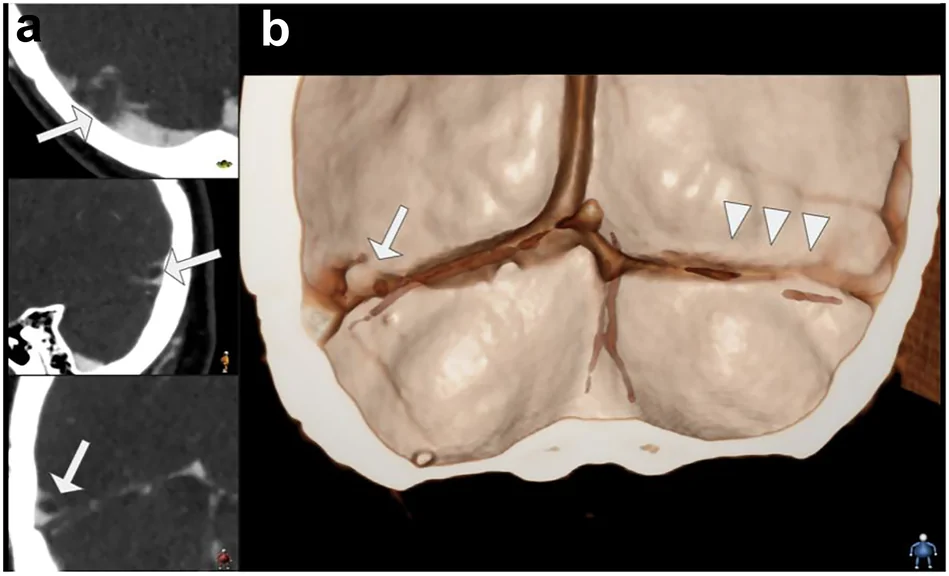
Transverse sinus stenosis in idiopathic intracranial hypertension. **a** Multiplanar reconstructions from contrast-enhanced CT venography (sagittal, coronal, axial) demonstrate bilateral transverse sinus narrowing: focal stenosis at the arachnoid granulation in the dominant right transverse sinus (arrows) and diffuse narrowing of the left transverse sinus (arrowheads), secondary to extrinsic elevated pressure or asymmetric sinuses. **b** Three-dimensional volume rendering confirms bilateral venous stenosis (arrows and arrowheads). These findings illustrate the association between venous stenosis and intracranial hypertension

Patients with idiopathic intracranial hypertension (IIH) may present with symptoms similar to those of CVT despite fundamentally different pathophysiology. These patients may exhibit venous stenosis without thrombosis, caused by external compression from elevated CSF pressure and bulging arachnoid granulations that protrude into the sinus lumen (Fig. 6) [30, 31]. Such venous stenoses can both result from and perpetuate elevated intracranial pressure, creating a self-reinforcing cycle. Systematic evaluation of venous sinus caliber should be performed in patients with headache consistent with IIH, as bilateral transverse sinus stenosis (or unilateral stenosis on the dominant side) may strengthen suspicion for IIH. Furthermore, systematic evaluation should include additional findings of elevated intracranial pressure: flattened posterior orbital globes, papilledema, perioptic CSF distension, widened Meckel’s cave, and pituitary gland flattening (empty sella).

### Hypoplastic sinus

A common anatomical variant is hypoplasia of one or more venous sinuses. The transverse sinuses frequently demonstrate asymmetric caliber, with one side smaller than the other. Aplasia or hypoplasia of the inferior sagittal sinus is also common, as illustrated in Sup Fig 14.

#### Technical pitfalls

**Fig. 7:**
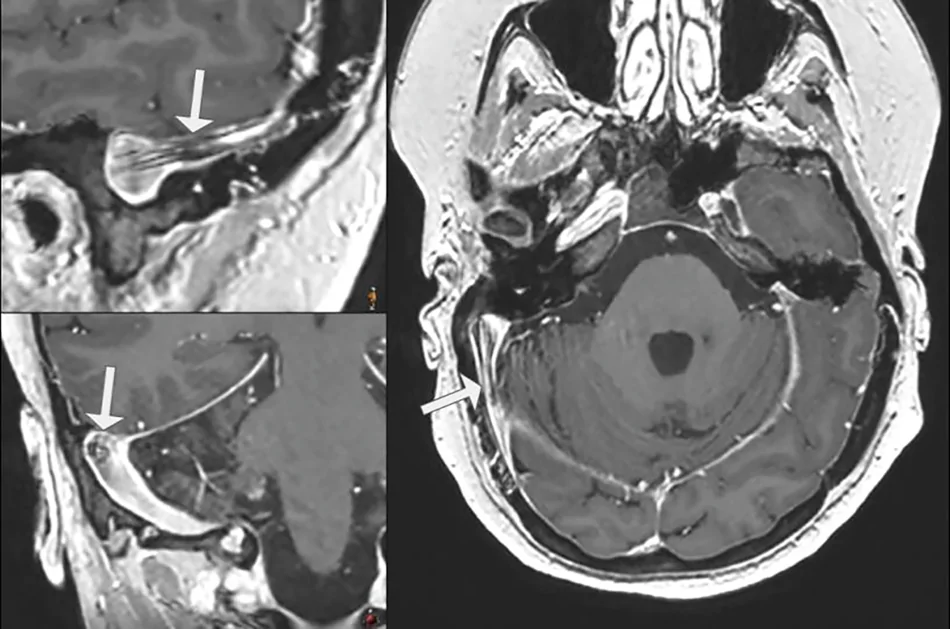
Flow artifact mimicking venous thrombosis. T1 3D GRE shows a signal void in the right transverse sinus (arrow) from hyperdynamic flow, which can mimic a filling defect or thrombus. Correlation with structural images, such as for example T2, is essential

**Fig. 8:**
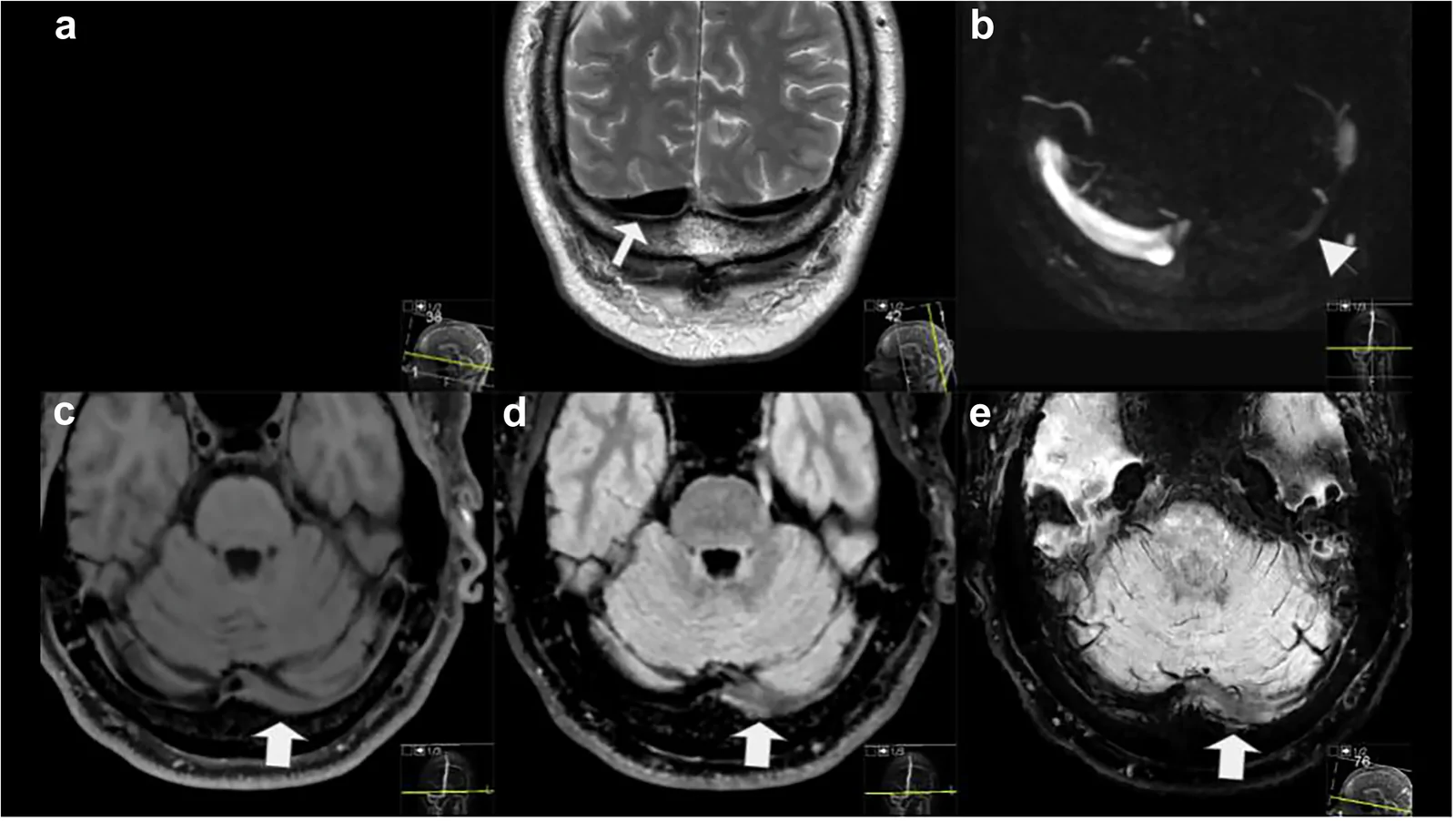
Slow flow mimicking thrombosis. Upper row: (**a**) T2 coronal with dark signal, due to loss of signal in high flow regions, most prominent in the right transversal sinus, and (**b**) phase contrast MRV shows apparent signal loss in the left transverse sinus (arrowhead), initially raising suspicion for thrombosis. Lower row: (**c**) T1 CUBE with fat saturation, (**d**) T2-FLAIR, and (**e**) SWI images with slight signal changes in the left transversal sinus (fat arrows)

**Fig. 9:**
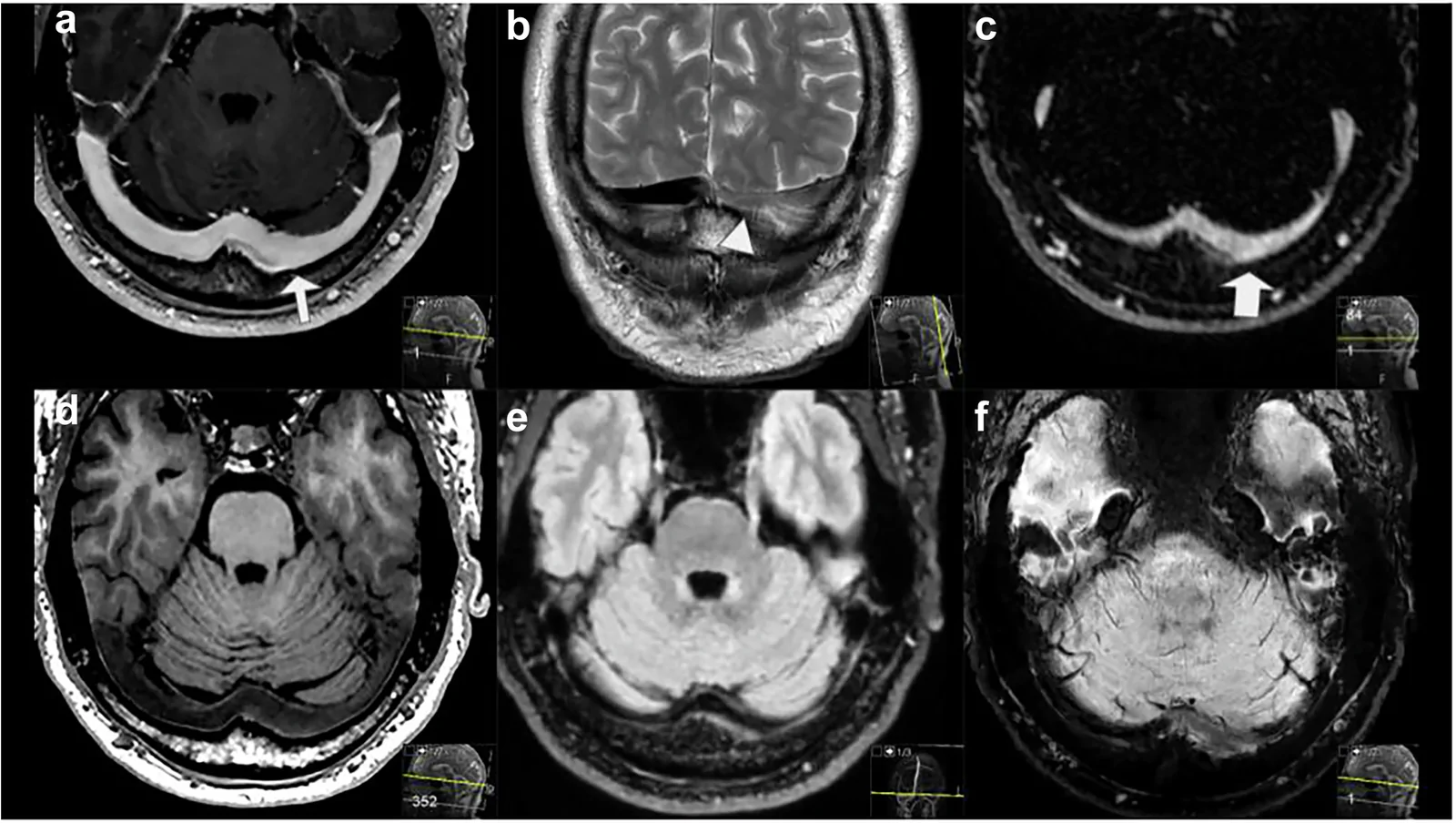
Follow-up excluding thrombosis. Upper row: **a** Post-contrast T1 showing total opacification (arrow), (**b**) T2 coronal with light signal changes (arrowhead), (**c**) time-resolved contrast-enhanced venography showing complete left transverse sinus opacification (fat arrow). Lower row: (**d**) T1, **e** T2-FLAIR, (**f**) SWI without signs of thrombus. Time-resolved MR venography confirms normal venous filling, ruling out thrombosis

False positives may result from timing artifacts when imaging is performed during arterial or early venous phases, before complete sinus opacification. Flow artifacts and contrast mixing (Fig. 7), in which unopacified blood enters contrast-enhanced vessels, can also mimic filling defects. Time-resolved MRA or multiphase CTA reduces these artifacts by demonstrating sequential venous filling from arterial through late venous phases (Sup Fig 15; Figs. 8 and 9).

**Fig. 10:**
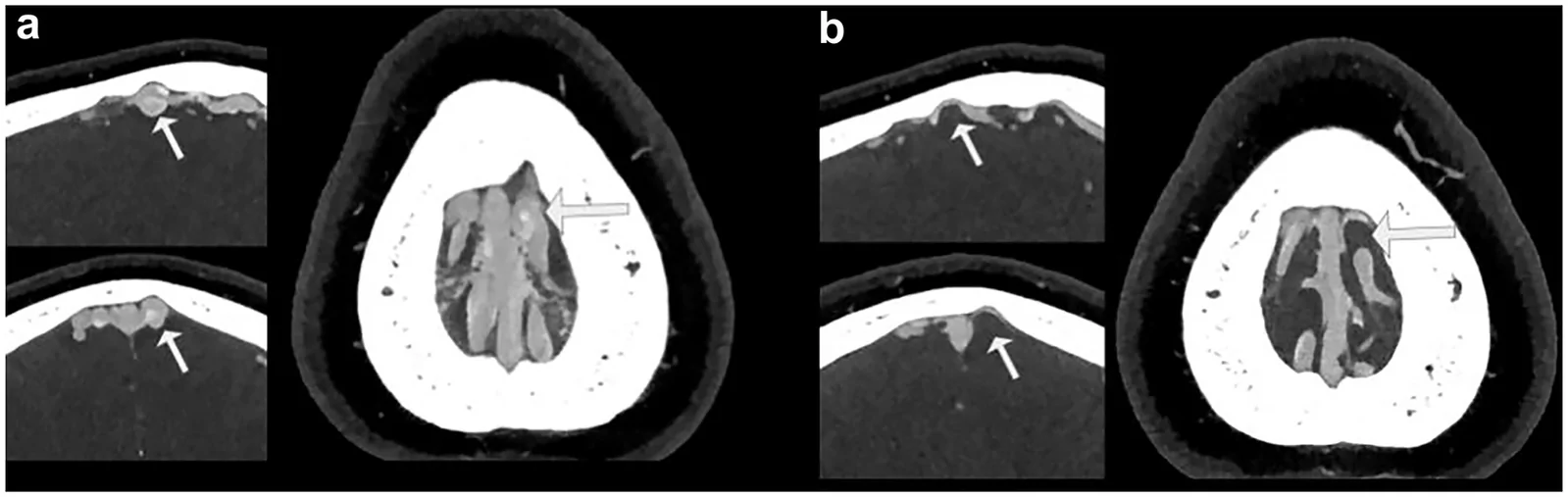
Venous changes in intracranial hypotension. **a** CT venography after dural puncture shows diffuse venous engorgement and absent arachnoid granulations (arrows). **b** Follow-up demonstrates normalized venous caliber with visible arachnoid granulations (arrows), indicating normalized intracranial pressure

Venography alone, without non-contrast imaging correlation, may misinterpret arachnoid granulations as thrombus [32]. Non-contrast CT should accompany contrast-enhanced venography to enable differentiation based on attenuation characteristics (Fig. 10).

Surgically ligated sinuses require correlation with operative notes.

The thrombus changes its appearance on CT and MRI depending on several factors, including timing. Acute thrombus (< 3 days) may appear isointense on T1-weighted imaging prior to methemoglobin formation, resulting in false-negative interpretation [33]. After the acute state, the thrombus increases its signal on T1-weighted MRI (Sup Fig. 16).

Non-contrast venography (TOF-MRV or phase-contrast imaging) readily demonstrates large venous structures but may show signal voids in slow-flow regions without thrombosis [33]. Multiphase CT or MR venography minimizes timing-related misinterpretation and prevents the misdiagnosis of completely opacified thrombosed sinuses on late-phase CT or T1-GRE sequences as patent [34].

Residual contrast enhancement in the dural venous sinuses following prior contrast administration may mimic the dense sinus sign and should not be mistaken for acute thrombosis.

### Cortical vein thrombosis

Cortical vein thrombosis occurs in small veins draining the cortex directly into bridging veins and subsequently into dural sinuses or larger medial and lateral superficial veins, including the veins of Labbé, Trolard and the superficial sylvian venous system. Limited collateral drainage pathways make cortical vein thrombosis particularly prone to cause direct venous stasis, resulting in ischemia and hemorrhage in the draining territory [35].

**Fig. 11:**
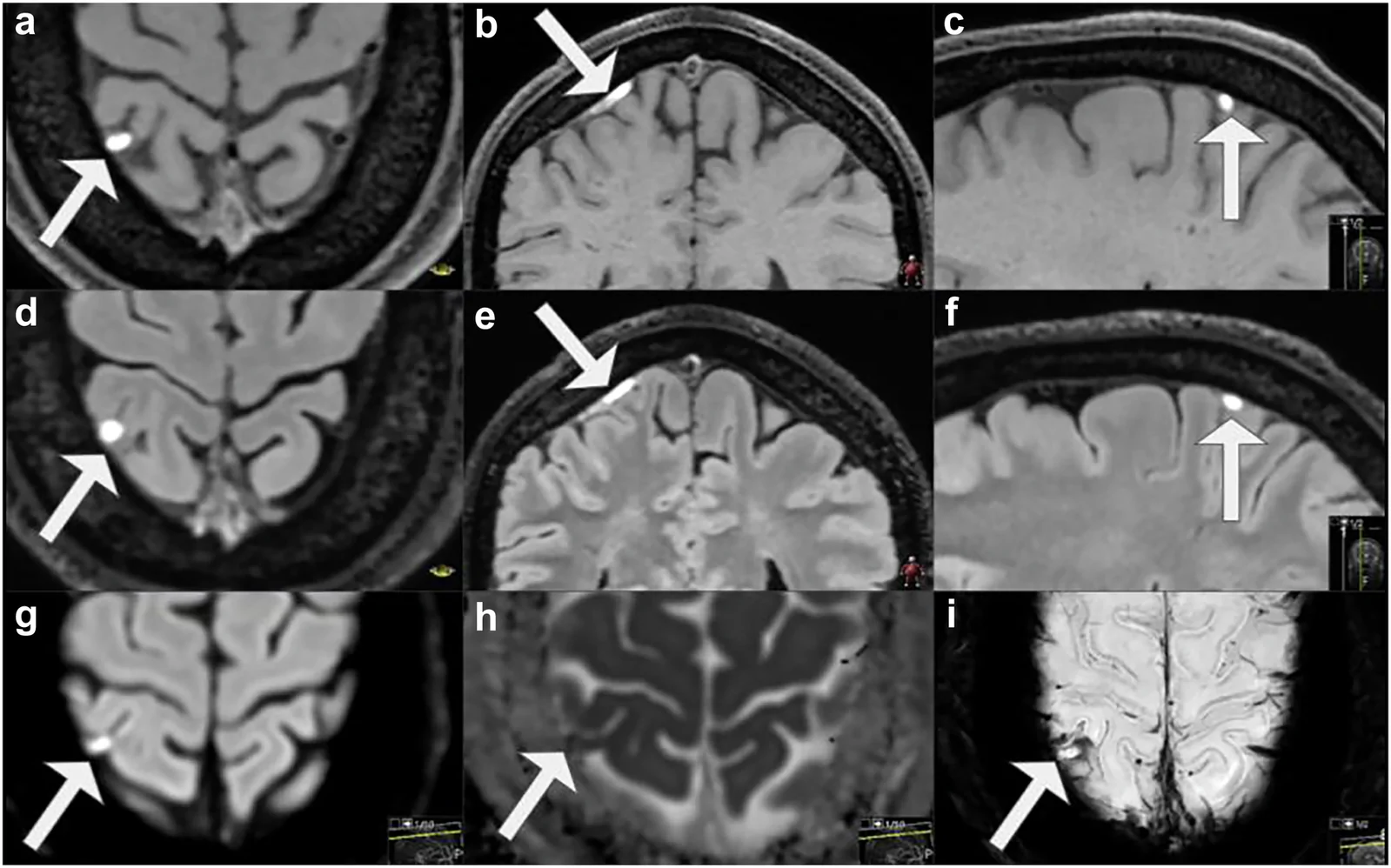
Right parietal cortical vein thrombosis on multisequence MRI. Upper row (**a**–**c**): T1-weighted images (axial, coronal, sagittal) show intrinsic hyperintensity in the thrombosed cortical vein (arrows). Middle row (**d**–**f**): T2-FLAIR (axial, coronal, sagittal) demonstrates hyperintense thrombus (arrows). Lower row: (**g**) DWI hyperintensity, (**h**) ADC hypointensity confirming restricted diffusion, and (**i**) SWI central hyperintensity (arrows), collectively confirming cortical vein thrombosis

Detection proves difficult on CT [36], given the small vein caliber and proximity to skull bone, where beam-hardening artifacts produce high-attenuating streaks near the bony interface. However, cortical edema, with or without ischemia or hemorrhage, should prompt careful examination of adjacent cortical veins. Comparison with contralateral cortical vein attenuation and signal intensity aids diagnosis. MRI sequences have variable presentation, with T2*/SWI [22, 36] and contrast-enhanced T1 being the most well studied [37]. SWI demonstrates venous stasis through increased conspicuity and tortuous veins in the affected region (Fig. 11).

Traumatic laceration may also produce thrombosis, leading to subsequent venous stasis in the tissue. CT with bone removal can increase conspicuity for venous structures close to the skull (Sup Fig. 17).

Mimics and pitfalls in the assessment of cortical vein thrombosis are numerous. Although subarachnoid hemorrhage may cause sulcal blooming on SWI, the appearance is less cord-like than that of a cortical venous thrombus. Thrombus signal evolves differently across MR sequences; SWI is more reliable in the early stage, while the T1 signal may remain elevated for a prolonged period beyond the hyperacute phase. Furthermore, the parenchymal complications associated with cortical vein thrombosis, including edema, hemorrhage, and infarction, are not specific and may arise from other ischemic, inflammatory, or hemorrhagic etiologies.

### Bridging vein thrombosis

The bridging veins drain blood from the cortical veins in the subarachnoid space, across the subdural space, and into the dural venous sinuses. Bridging vein thrombosis rarely occurs in isolation; instead, it typically forms part of widespread intracranial venous thrombosis involving dural sinuses, cortical veins and the intervening bridging veins. These thrombi can be present in traumatic subdural hematomas, mostly reported in a pediatric population in the context of abusive head trauma [38], with limited data available in adults, warranting careful evaluation for subtle bridging vein thrombosis in trauma patients presenting with subdural hemorrhage (Sup Fig. 18).

Mimics and pitfalls in the assessment of bridging vein thrombosis are few but important. One challenge is distinguishing the small venous structures from adjacent hyperattenuating cortical bone. Acute subdural hematoma may mimic bridging vein thrombosis, but does not produce the cord-like structure of a thrombosed vein. When an extraaxial hemorrhage is colocalized with a potential thrombus, the causal relationship is often difficult to establish. Thrombosed intracranial venous lakes may also mimic bridging vein thrombosis, but do not extend into the SSS, as cortical veins can. Finally, bridging veins are frequently partially compressed by arachnoid granulations, which should not be mistaken for thrombus; correlation between non-contrast and contrast-enhanced CTV, together with consideration of intracranial pressure, aids in this assessment.

### Intracranial venous lake thrombosis (IVLT)

**Fig. 12:**
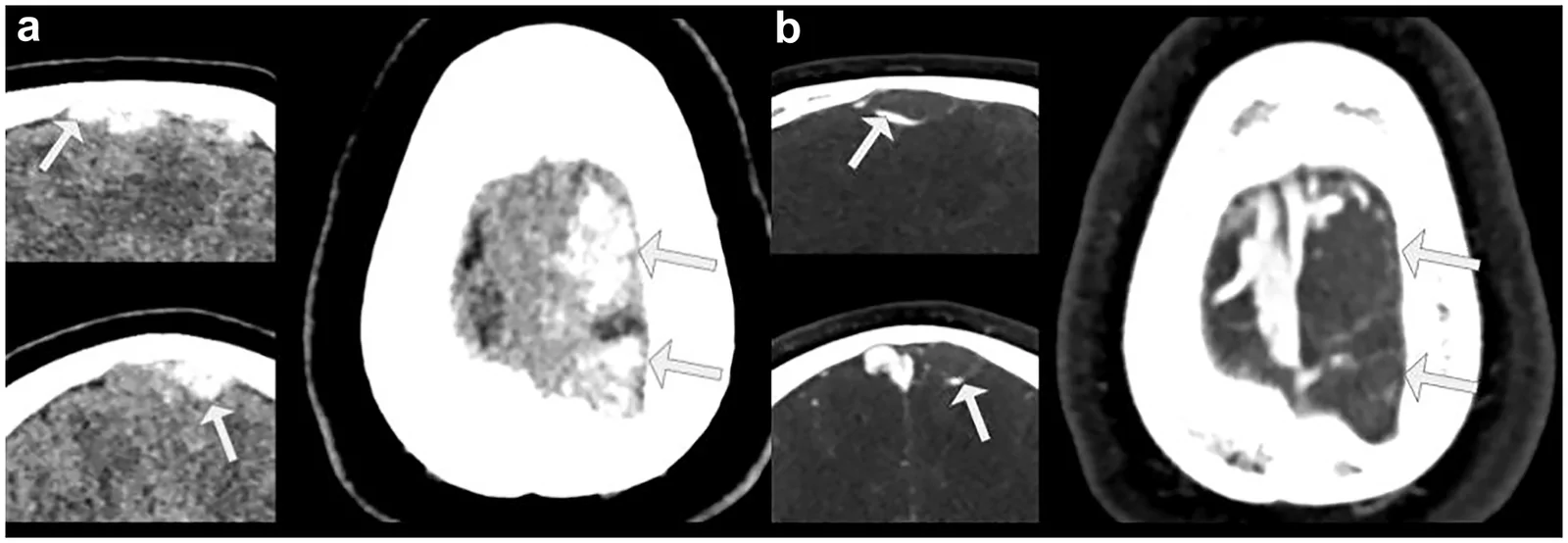
Intracranial venous lake thrombosis (IVLT). **a** Non-enhanced CT shows hyperattenuating parasagittal structures at the lateral venous lakes (arrows). **b** CT venography demonstrates absent opacification (arrows), confirming IVLT

Intracranial venous lake thrombosis represents a recently described entity affecting lateral venous lakes adjacent to the SSS. It predominantly occurs in patients with intracranial hypotension and prothrombotic states, classically presenting with postural headache in postpartum women [39]. Unlike cortical veins, these lakes receive drainage from diploic and meningeal vessels rather than brain parenchyma, and are normally thin structures filled with arachnoid granulations near the skull convexity (Fig. 12) [40].

IVLT manifests in patients with findings of intracranial hypotension, including diffusely engorged intracranial veins and enlarged lateral lakes containing thrombotic material. The characteristic “pancake-like” appearance distinguishes venous lakes from the cord-like morphology of cortical or bridging veins. IVLT may mimic an extraaxial hematoma, cortical vein or bridging vein thrombosis. This parasagittal location may also harbor dural AV-fistulas [41], and convexity meningiomas commonly arise from arachnoid cap cells concentrated at the lateral venous lacunae along the SSS [40]; tumors in this location may consequently impair regional venous drainage or cause partial or complete obstruction of the SSS. Venous lakes are normally filled with arachnoid granulations (Sup Fig. 19).

Mimics and pitfalls in the assessment of IVLT are few and mostly related to intracranial pressure and CSF volume. Although not extensively studied, no cases of IVLT without concomitant findings of intracranial hypotension have been reported. In normal intracranial pressure, the venous lakes are thin and closely apposed to the inner table of the skull. Arachnoid granulations protruding into the venous lakes should not be mistaken for thrombosis. In intracranial hypotension, dilation of the lakes likely reflects shrinkage of the arachnoid granulations, and this distension may promote clot formation, particularly in the presence of additional prothrombotic factors such as the postpartum state or a coagulation disorder. Cortical and bridging vein thrombosis share the parasagittal location but are distinguished by their tubular morphology. Convexity meningiomas, which arise preferentially from arachnoid cap cells at the lateral venous lacunae, may cause secondary venous lake obstruction; venography will demonstrate extrinsic compression rather than primary thrombosis. Convexity subdural and epidural hematomas are potential mimickers but do not localize exclusively to the parasagittal venous lakes.

### Central venous thrombosis

Central or deep CVT represents a frequently devastating clinical condition. Impaired drainage from deep structures, including the thalami, combined with minimal collateral pathways, produces rapid edema formation, blood-brain barrier disruption, hypoxic perfusion changes, and hemorrhage. Thrombus propagation into the vein of Galen and the straight sinus further exacerbates disease severity. Patients typically present with a progressive impairment of consciousness ranging from delirium to coma, with frequent seizures.

**Fig. 13:**
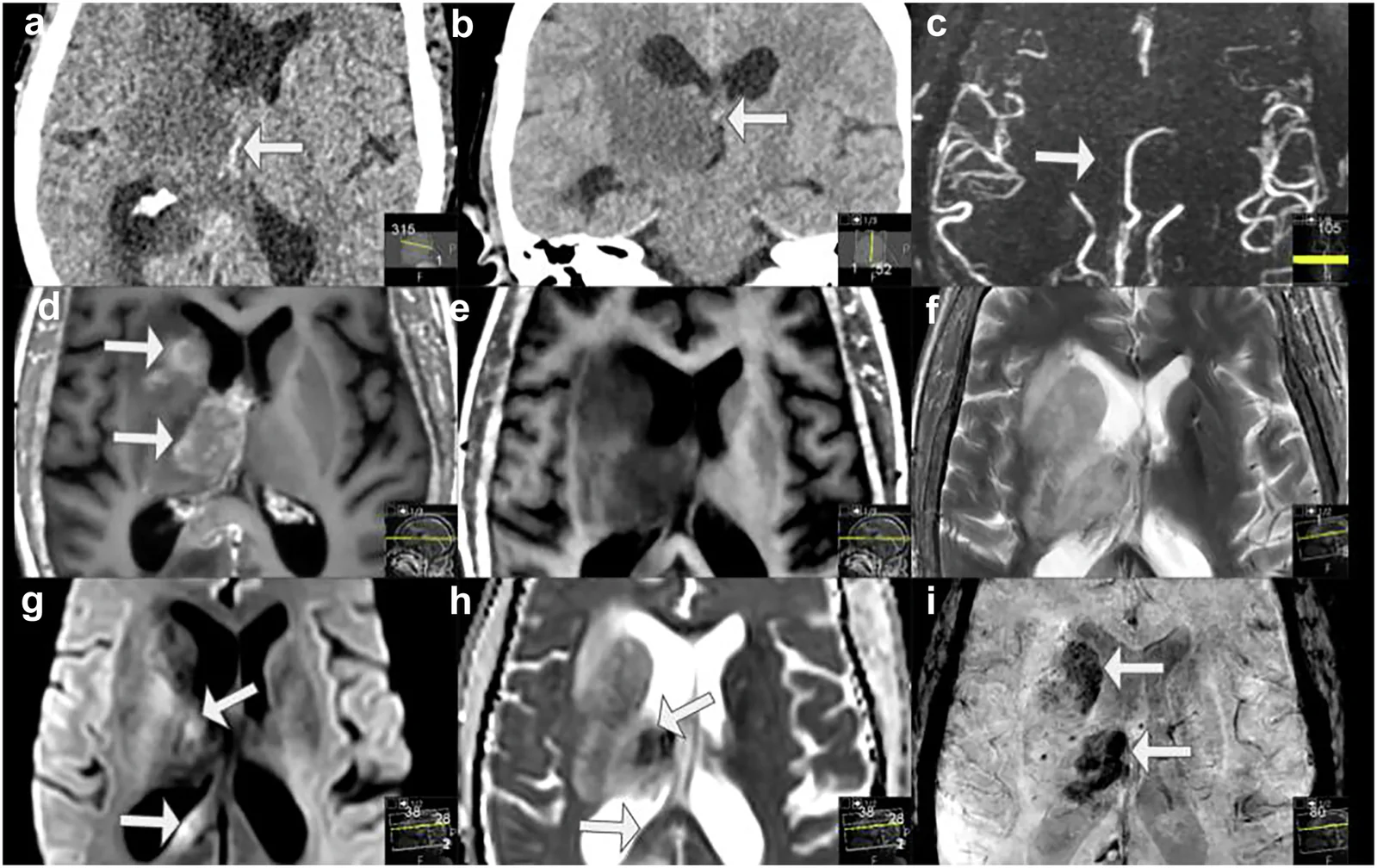
Right internal cerebral vein thrombosis with venous infarction mimicking malignancy. Upper row: **a** Non-enhanced axial CT and (**b**) coronal CT show hyperattenuating right internal cerebral vein (arrows) with central hemispheric edema. **c** CT venography demonstrates absent opacification of the right internal cerebral vein (arrow), confirming thrombosis. Middle row: **d** Non-enhanced T1 and (**e**) post-gadolinium T1 show enhancement in deep gray nuclei (arrows), with (**f**) T2-weighted imaging demonstrating surrounding edema. Lower row: (**g**) DWI and (**h**) ADC map show patchy restricted diffusion (arrows) indicating cytotoxic edema, and (**i**) SWI demonstrates signal loss from hemorrhagic transformation (arrows). This constellation of findings mimicked malignancy (lymphoma or glioma), but biopsy confirmed venous infarction secondary to unilateral deep venous thrombosis

Asymmetric involvement affecting only one hemisphere creates a diagnostic challenge. Unilateral edema and blood-brain barrier breakdown may closely simulate malignancy, particularly lymphoma or high-grade glioma (Fig. 13) or infarction. Careful scrutiny of internal cerebral veins, venographic imaging, and perfusion studies demonstrating hypoperfusion patterns is helpful for accurate diagnosis and avoiding misinterpretation.

Mimics and pitfalls in the assessment of central or deep CVT primarily arise from the many conditions that can produce a similar pattern of deep hemispheric and thalamic signal change. When considering arterial causes, occlusion of the artery of Percheron produces bilateral thalamic infarction but typically results in a smaller, more medial volume of injury. For inflammatory, infectious, and autoimmune etiologies, the absence of thrombus in the central or deep cerebral veins aids the differential diagnosis.

### Thrombus in the inferior anastomosing vein of Labbé

The inferior anastomotic vein of Labbé drains the lateral temporal and parietal cortex into the transverse sinus. It anastomoses with the superior anastomotic vein of Trolard and the superficial middle cerebral vein. Thrombosis of the inferior draining vein of Labbé (Sup Fig. 20) produces a characteristic pattern of posterior temporal lobe affectation, typically hemorrhage with vasogenic and cytotoxic edema. Posterior temporal lobe lesions with edema or hemorrhage should prompt systematic evaluation for vein of Labbé thrombosis.

The most important consideration in the vein of Labbé thrombosis is to suspect the diagnosis when parenchymal complications affect the posterior temporal lobe in one hemisphere. The thrombus itself may be less conspicuous due to its proximity to the skull bone, but on direct inspection, a parenchymal hemorrhage in the temporal lobe may be associated with an acute thrombus in the vein of Labbé.

### Cavernous sinus thrombosis

The cavernous sinus is a paired dural venous sinus located on each side of the sella turcica, connected by intercavernous sinuses anteriorly and posteriorly. It receives drainage from the superior and inferior ophthalmic veins and from the superficial middle cerebral vein via the sphenoparietal sinus. It drains posteriorly via the superior and inferior petrosal sinuses and inferiorly to the pterygoid venous plexus below the skull base. Cavernous sinus thrombosis presents with orbital venous congestion producing erythematous globes and proptosis, impaired visual acuity, ocular motor impairment and pain. Clinically, a carotid-cavernous fistula, most commonly caused by trauma, is an important differential diagnosis, and a distinctive feature of cavernous sinus thrombosis is contralateral spread via the intercavernous sinuses within 24–48 h [42].

Ongoing infection spreading from facial or paranasal sinus sources represents the most common etiology, fundamentally altering imaging characteristics. Unlike other intracranial thromboses, cavernous sinus thrombus may consist of infectious debris or purulent material rather than organized clot, and may thus lack typical hyperattenuation on CT or T1 hyperintensity on MRI. Common imaging features of cavernous sinus thrombosis include cavernous sinus bulging and reduced internal carotid artery caliber. Dilated superior ophthalmic veins provide an important secondary sign of impaired venous drainage (Sup Fig. 21) [43].

Standard 20-s venous-phase imaging is inadequate for cavernous sinus evaluation due to slower filling kinetics. A minimum 45-s delay is necessary for optimal opacification and accurate assessment [43].

### Miscellaneous cases

Prominent intramedullary veins on SWI may reflect various pathologies beyond thrombosis, requiring careful differential diagnosis. Infectious etiologies, particularly COVID-19, can produce microthrombi within these vessels (REF). In contrast, dural arteriovenous fistulas demonstrate similar SWI appearance due to venous congestion from arteriovenous shunting without actual thrombosis (Sup Fig. 22). The distinction is made angiographically: AV-fistulas show early venous filling during the arterial phase, whereas thrombosis shows venous occlusion. Table 3 summarizes clinical symptoms and radiological findings based on thrombous location.

**Table 3:** Clinical symptoms, complications, and associated radiological findings in intracranial venous thrombosis

Location of thrombosis	Clinical symptoms	Acute and subacute radiological findings	Delayed radiological findings
Dural venous sinus	Headache, visual changes, focal neurological deficits	Vasogenic edema, increased intracranial pressure, cytotoxic edema, hemorrhage	Hydrocephalus, incomplete recanalization, thrombus propagation, dural arteriovenous fistula, gliosis, recurrence
Cortical vein	Headache, seizure, focal neurological deficits	Subarachnoid hemorrhage, vasogenic edema, cytotoxic edema	Gliosis in infarcted areas
Bridging vein	Headache	NA	NA
Intracranial venous lake thrombosis	Headache, initially postural	NA	NA
Central vein	Altered mental status, decreased level of consciousness, coma, seizures	Vasogenic edema, increased intracranial pressure, cytotoxic edema	Hemorrhage, thrombus propagation. Gliosis in infarcted areas
Inferior Anastomosing vein	Headache, aphasia, hemianopia	Vasogenic edema, cytotoxic edema	Hemorrhage, thrombus propagation. Gliosis in infarcted areas.
Cavernous sinus	Headache, fever, sepsis, chemosis, periorbital edema, cranial nerve symptoms, loss of vision, eye pain	Intraorbital edema, optic papilledema and thrombosis of the superior ophthalmic vein	Meningitis, intracranial infectious spread

Leukostasis in leukemia represents another distinct mechanism affecting medullary veins. Centripetal hemorrhage in patients with marked leukocytosis and thrombocytopenia should raise suspicion for leukostasis-induced bleeding from impaired medullary venous flow (Sup Fig. 23).

## Complications and associated findings

### Imaging strategy: recommended protocols

Both CT with CTV and MRI with MRV are reliable modalities for diagnosing or excluding intracranial venous thrombosis, and either can be used as the primary imaging strategy. The choice between them is guided by clinical context, patient factors, and local availability rather than diagnostic superiority. CT represents the most readily available acute imaging modality, with CT venography providing a reliable assessment of cerebral venous patency, but it requires appropriate timing with good venous filling to avoid false positive interpretation. However, MRI offers several advantages: superior characterization of cerebral complications, such as differentiating vasogenic from cytotoxic edema, visualization of venous stasis and thrombus, and avoidance of radiation exposure. Additionally, MRI can assess blood flow within venous structures without contrast administration using non-contrast techniques, which is advantageous in pregnancy, where gadolinium-based contrast agents are generally avoided [44]. A decision-oriented flowchart summarizing the choice between CT and MRI is provided in Sup Fig. 24.

Three-dimensional T1-weighted black-blood imaging shows high sensitivity and specificity for visualizing subacute venous thrombosis, including cortical vein thrombosis [45, 46]. Contrast-enhanced 3D MPRAGE sequences demonstrate high accuracy for identifying CVST, with a sensitivity of 91% and a specificity of 98%. High-resolution 3D T2-SPACE offers comparable or superior performance without contrast administration and may be particularly useful when gadolinium is contraindicated [47]. Non-contrast time-of-flight MR venography demonstrates reasonable sensitivity (80%) but limited specificity (65%) in diagnosing CVST [48]. Radiologists should therefore combine TOF with complementary sequences including T1, T2/T2-FLAIR, SWI, DWI, and contrast-enhanced techniques. A multisequence approach combining 3D phase-contrast MRV, T1- and T2-weighted images with non-enhanced CT achieves near-perfect diagnostic accuracy for CVST [33]. However, non-contrast CT and CTV are also a feasible alternative in pregnant women, more often easy to interpret and with a negligible dose of radiation to the fetus [32, 49]. Technical aspects of MRI and CT imaging in CVT are further detailed in a previous review by Sadik et al [23].

### Clinical implications and reporting

Cerebral venous thrombosis poses a significant diagnostic challenge due to its diverse etiologies, variable presentations, and symptom overlap with other pathologies. Radiologists must maintain systematic vigilance for venous abnormalities in every neuroimaging study, as clinical diagnosis is often elusive.

When thrombosis is identified, two critical distinctions guide management. First, determine whether the thrombus is acute or chronic. Acute thrombosis warrants anticoagulation and close monitoring, while chronic occlusion may represent an incidental finding. Second, characterize complications clearly and systematically. Differentiate edema from ischemia using T2-weighted and diffusion-weighted sequences, and document hemorrhagic complications (notably, venous hemorrhage does not contraindicate anticoagulation).

Clinically, most patients with mild symptoms are successfully managed with anticoagulation (LMWH or heparin). For patients with altered mental state, coma or severe neurologic symptoms, radiologic assessment should include evaluation of mass effect requiring urgent decompressive surgery. Hemicraniectomy or bilateral craniectomy may prove lifesaving in cases of severe cerebral swelling from venous stasis, providing temporizing treatment while anticoagulation takes effect [1, 8, 50]. In patients with severe clinical symptoms that do not respond to anticoagulation, endovascular treatment may be warranted to achieve immediate venous decompression and facilitate subsequent anticoagulation by reducing thrombus burden [1, 51].

Follow-up studies may demonstrate complete recanalization or persistent chronic luminal changes. To ensure comprehensive communication with referring clinicians and facilitate optimal patient management, radiological reports should systematically address the following elements:

**Structured reporting template:**

- Location of thrombosis (specific sinuses and/or veins affected)
- Extent of thrombosis (partial vs. complete occlusion)
- Age of thrombus (acute, subacute, or chronic, based on imaging characteristics)
- Parenchymal complications (presence and extent of edema, hemorrhage, venous infarction)
- Collateral venous drainage pathways (present or absent)
- Mass effect and risk of herniation (if present)
- Associated findings (infectious source, skull fracture, underlying malignancy, signs of intracranial hypertension or hypotension)
- Comparison with prior studies (if available: progression, stability, or recanalization)
- Recommendations for additional or follow-up imaging and timing

If no thrombosis is identified in patients with suspected CVT, systematic evaluation for alternative diagnoses becomes critical. Idiopathic intracranial hypertension (IIH) is an important differential diagnosis, with significant overlap in both demographics and clinical presentation [52].

### Future outlooks—AI

Diagnostic accuracy for intracranial venous thrombosis has improved substantially with faster imaging acquisition, higher resolution, advanced CT detectors, and sophisticated MR sequences, including 3D imaging, time-resolved venography, and non-contrast techniques. Artificial intelligence algorithms show promise for further enhancing diagnostic accuracy [53] through automated venous system segmentation, detection of filling defects, and identification of secondary findings, such as parenchymal complications [54]. AI-based detection may prove particularly valuable for CVT given the propensity for subtle findings near the skull to be overlooked on visual inspection [12]. However, validation in large, diverse patient populations and integration into clinical workflows remain necessary before widespread implementation.

## Conclusion

Accurate diagnosis of CVT fundamentally impacts patient outcomes and requires systematic recognition of location-specific imaging patterns. Radiologists must assess for acute thrombosis and associated complications, employing optimal protocols including multiphase or time-resolved venography interpreted alongside structural sequences to characterize thrombus evolution, morphology, and parenchymal complications. A methodical evaluation strategy combined with awareness of common mimics and pitfalls enhances diagnostic accuracy and ultimately improves clinical outcomes.

## Supplementary information

ELECTRONIC SUPPLEMENTARY MATERIAL

## Data Availability

Not applicable. This is an educational review article and does not report original research data.

## Abbreviations

CT: Computed tomography
CTV: Computed tomography venography
CVST: Cerebral venous sinus thrombosis
CVT: Cerebral venous thrombosis
IVLT: Intracranial venous lake thrombosis
MRI: Magnetic resonance imaging
MRV: Magnetic resonance venography
SSS: Superior sagittal sinus
