# Supplementary material for: Intracranial venous thrombosis imaging by anatomical location: an educational review with mimics and diagnostic pitfalls
Source: Insights Imaging. 2026 Sep 18;17:230. doi: 10.1186/s13244-026-02381-7 (PMC13588991; doi:10.1186/s13244-026-02381-7)
Supplement: Supplementary file 1 [file 13244_2026_2381_MOESM1_ESM.pdf]

Intracranial venous thrombosis imaging by anatomical location: An educational review with mimics and diagnostic pitfalls

Electronic supplementary material

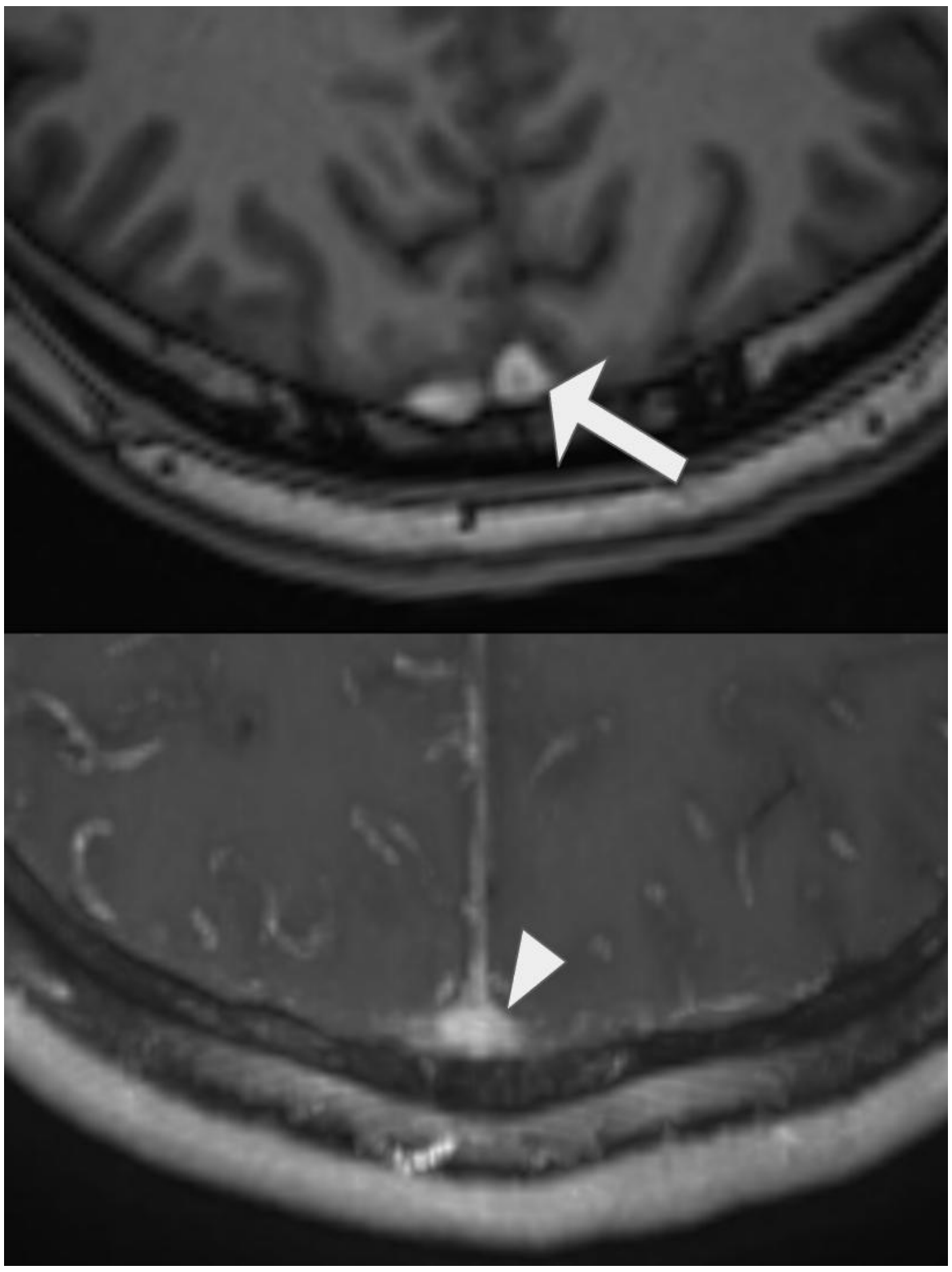

Supl Fig 1: *Pitfall in imaging after gadolinium contrast injection. (a) Non-enhanced T1-weighted image demonstrates intrinsic hyperintensity within the superior sagittal sinus (arrow), consistent with subacute thrombus. (b) Post-gadolinium T1-weighted image in the parenchymal phase shows complete opacification of the sinus (arrowhead), mimicking a patent vessel. This case illustrates a critical pitfall: without prior review of the non-enhanced T1 sequence, the hyperintense thrombus may be obscured by the gadolinium enhancement, resulting in a false-negative interpretation.*

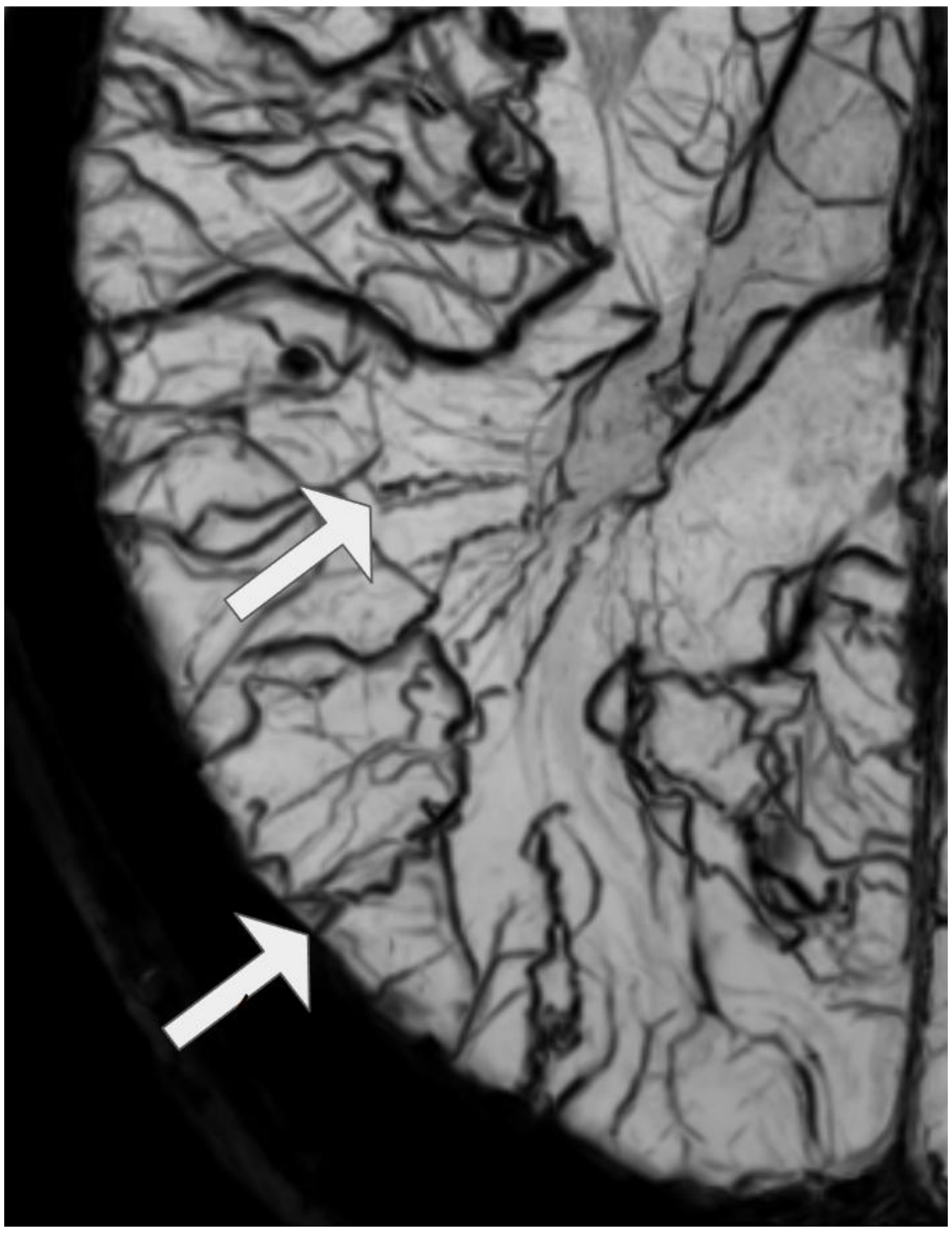

Supl Fig 2: *Venous congestion secondary to right transverse sinus thrombosis. Axial susceptibility-weighted imaging (SWI) shows dilated, curvilinear deep and superficial medullary veins with corkscrew appearance in the right hemisphere (arrows), reflecting increased venous pressure and impaired drainage.*

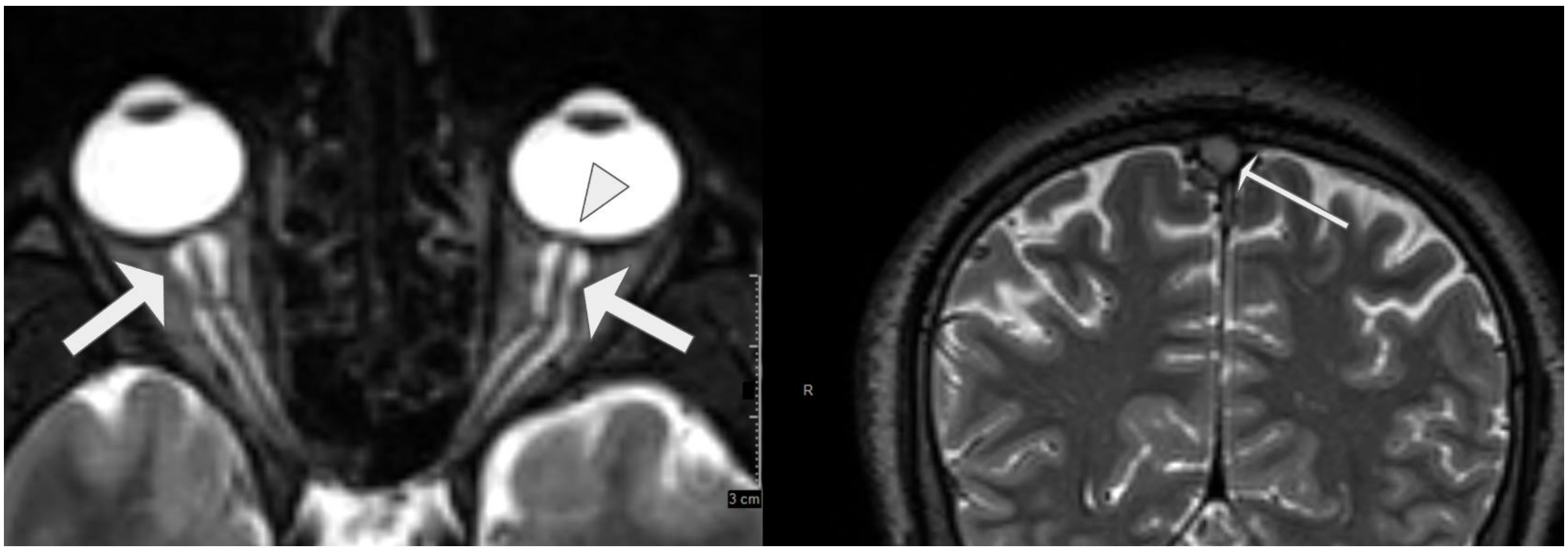

Supl Fig 3: *Coexisting manifestations of increased intracranial pressure alongside superior sagittal sinus thrombosis. (a) Axial 3D T2-weighted imaging reveals bilateral expansion of the perioptic cerebrospinal fluid space (arrows) and protrusion of the left optic nerve head (arrowhead), indicative of papilledema. (b) Coronal T2-weighted sequence displays an absent flow void within the superior sagittal sinus (thin arrow), corresponding to intraluminal thrombus.*

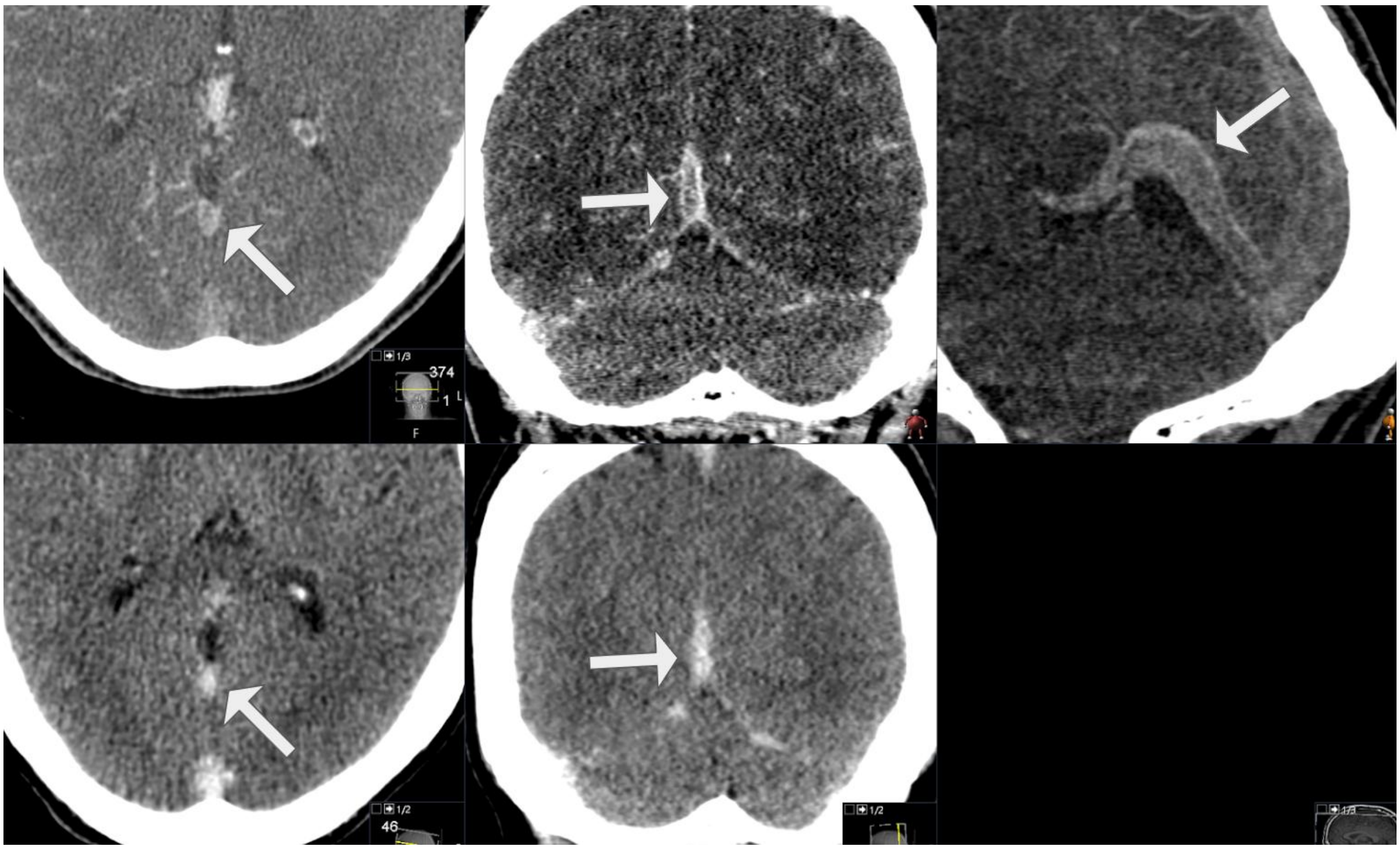

Supl Fig 4: *Straight sinus and vein of Galen thrombosis. Upper row: CT venography in (a) axial, (b) coronal, and (c) sagittal planes demonstrates filling defects delineated by thin peripheral contrast in the straight sinus and vein of Galen (arrows). Lower row: Non-enhanced CT in (d) axial and (e) coronal planes shows hyperattenuating thrombus in corresponding locations (arrows), confirming acute thrombosis.*

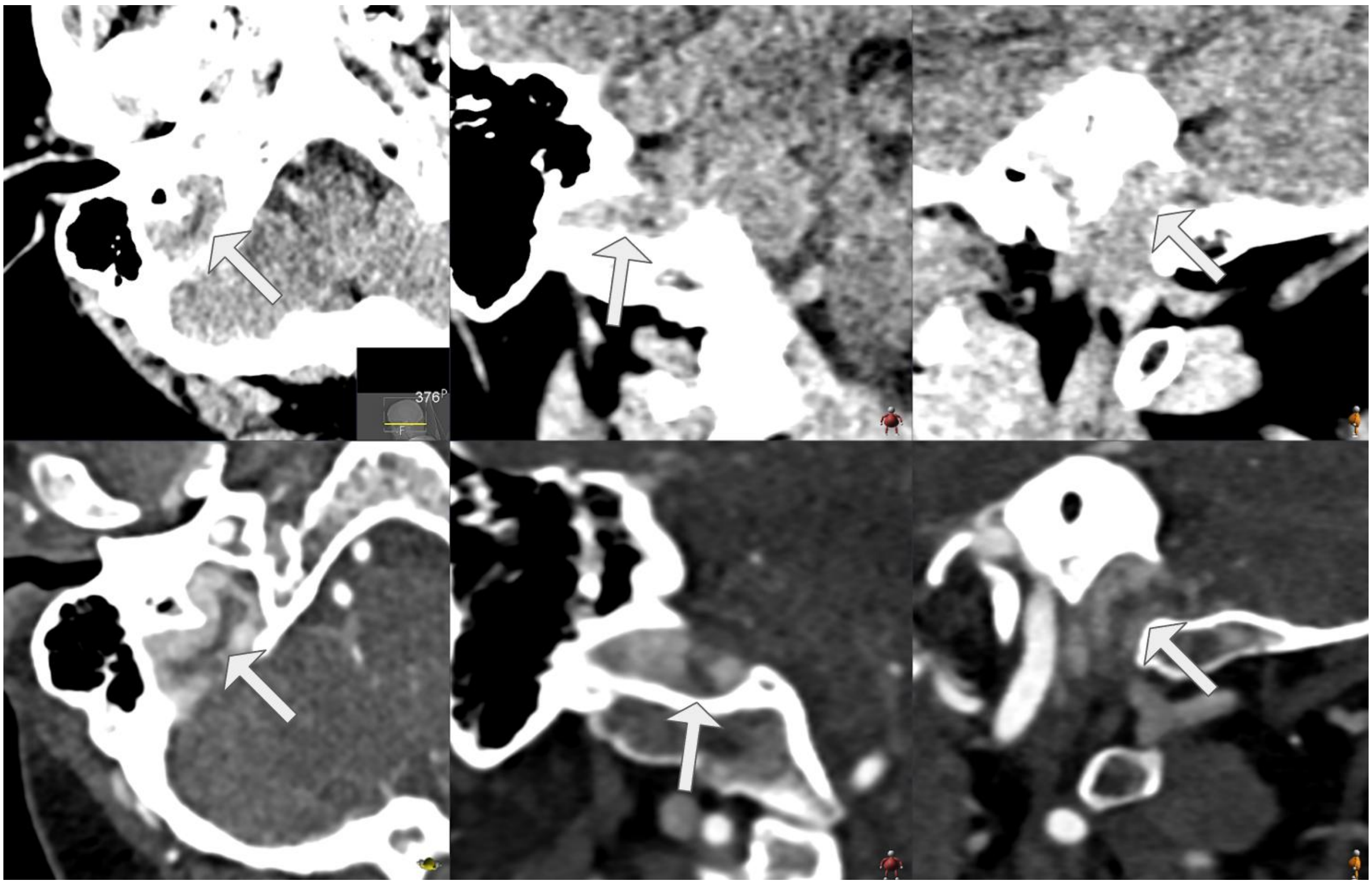

Supl Fig 5: *Chronic sigmoid sinus thrombosis. (a-c) Non-enhanced CT demonstrates heterogeneous attenuation with low-attenuation areas (arrow), indicating chronic organized thrombus in the right sigmoid sinus. (d-f) CT venography shows irregular filling, with central recanalization (arrow).*

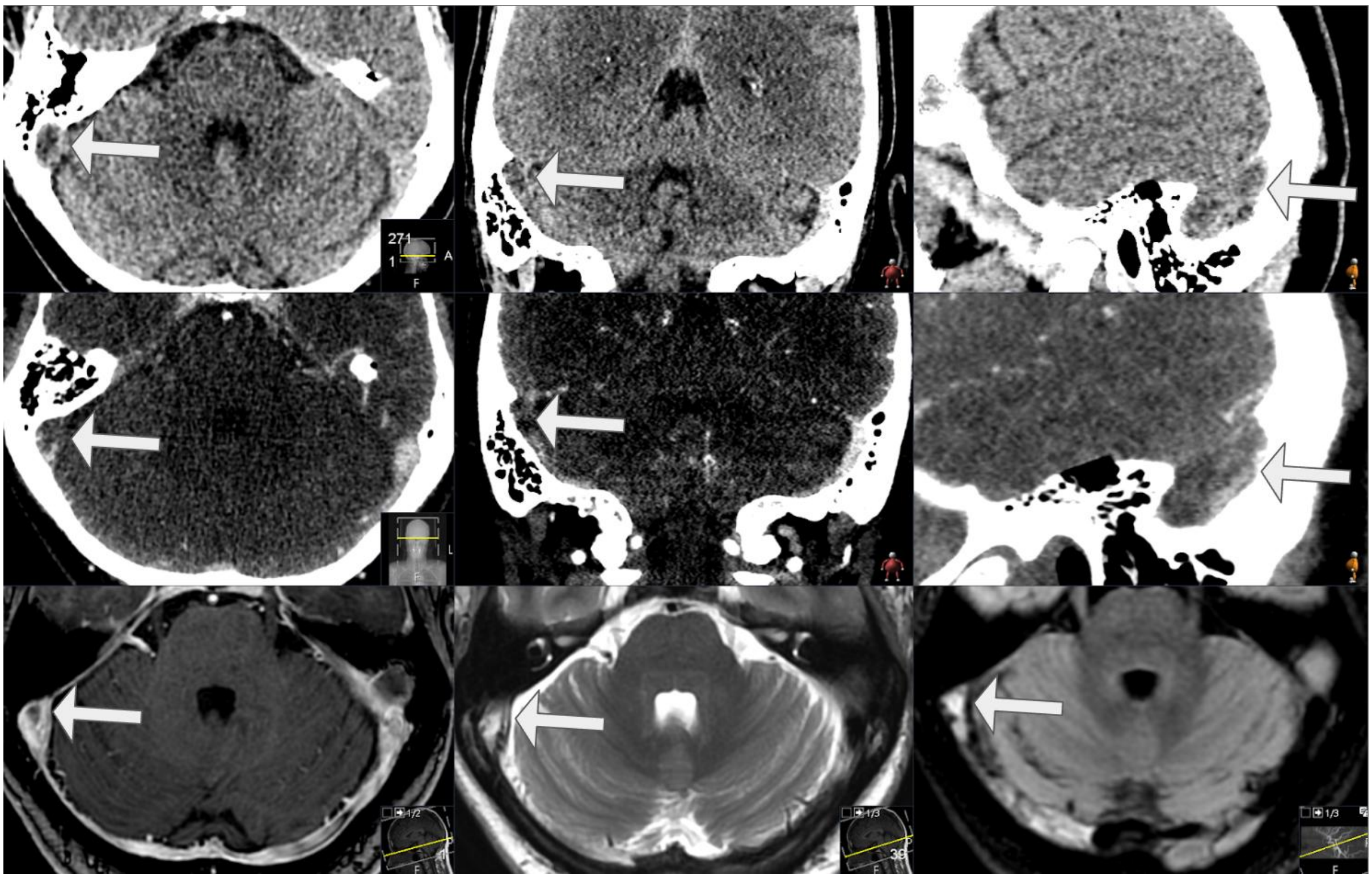

Supl Fig 6: *Chronic right transverse and sigmoid sinus thrombosis. Upper row: (a–c) Non-enhanced CT (axial, coronal, sagittal) shows hypoattenuating organized thrombus in the right transverse and sigmoid sinuses (arrows). Middle row: (d–f) CT venography (axial, coronal, sagittal) demonstrates incomplete opacification compared to normal left transverse and sigmoid sinuses (arrows). Lower row: (g) T1 post-gadolinium with possible enhancement of organized thrombus. The thrombus was not hyperintense on non-enhanced T1 (not shown), consistent with chronic rather than subacute thrombosis. Parenchymal phase contrast-enhanced sequences may obscure venous thrombosis as chronic thrombi can demonstrate delayed enhancement, mimicking patent sinuses. (h) T2 and (i) T2-FLAIR showing hyperintense signal with loss of flow void (arrows).*

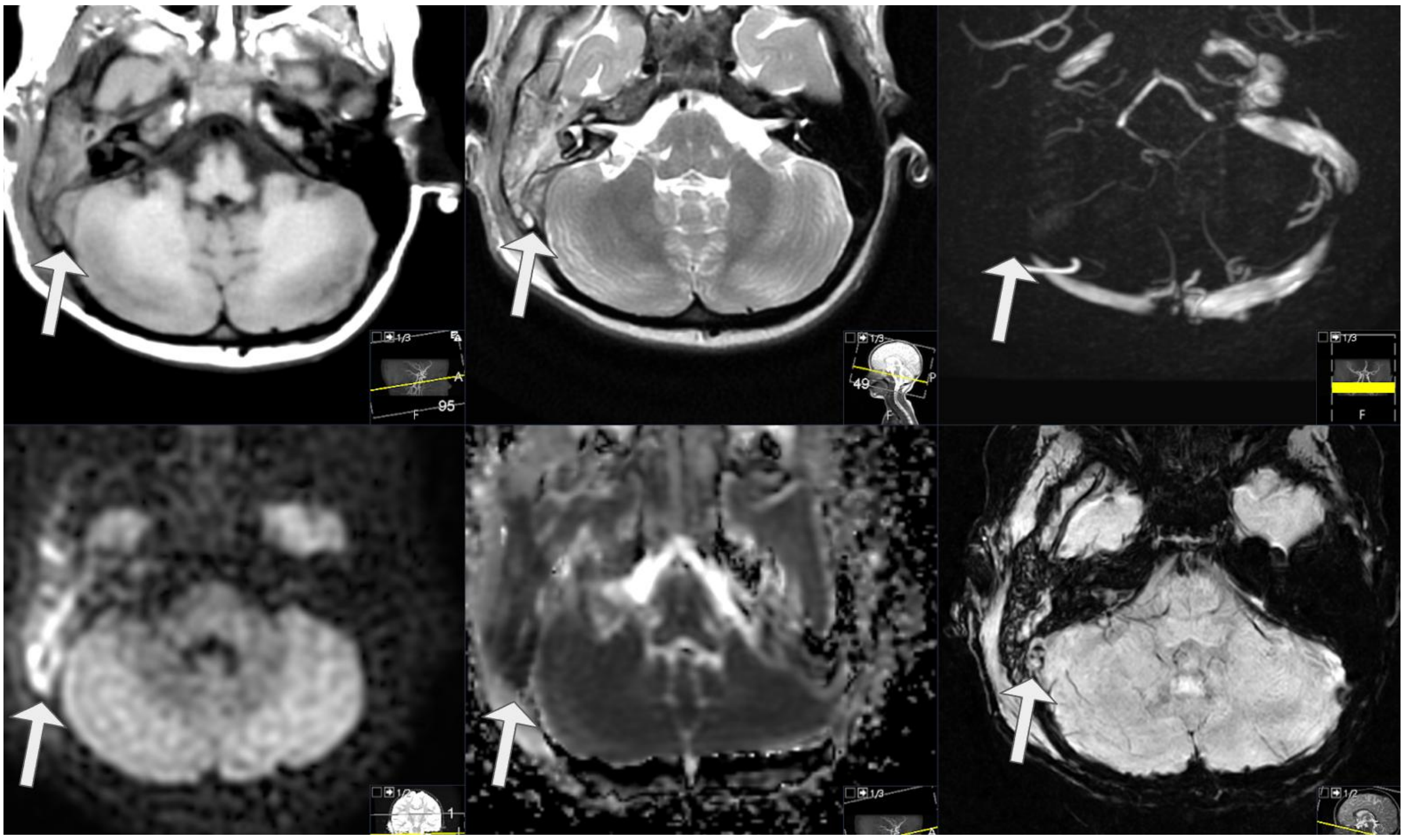

Supl Fig 7: *Septic sigmoid sinus thrombosis from otomastoiditis. Upper row: (a) T1 and (b) T2 show isointense sinus (arrows) with mastoid inflammation. (c) TOF-MRV demonstrates absent flow (arrow). Lower row: (d) DWI hyperintensity, (e) Low ADC in mastoid and sinus (arrows), (f) SWI heterogeneous signal (arrow).*

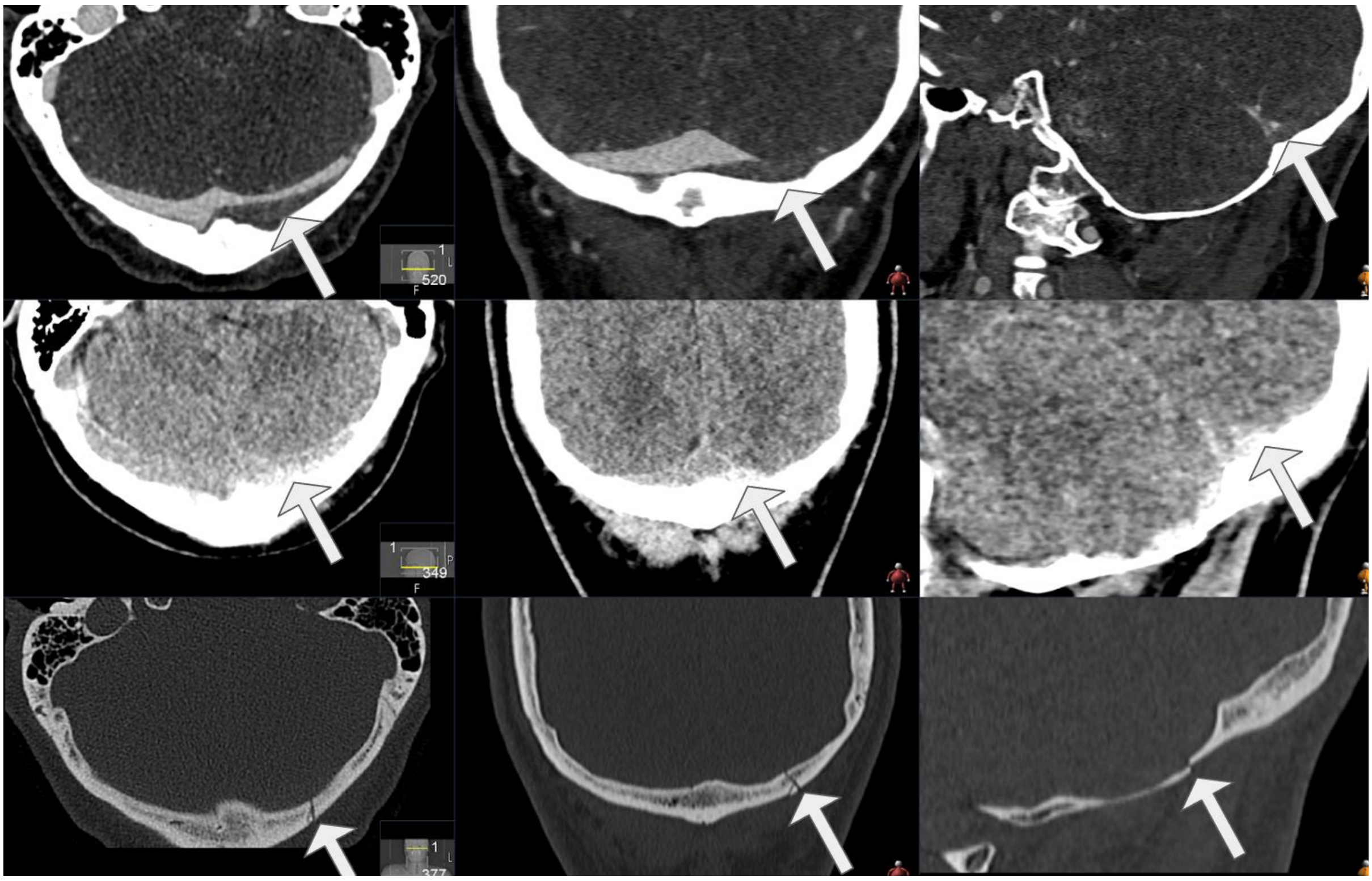

Supl Fig 8: *Traumatic left transverse sinus compression. CT venography (upper row) shows focal sinus narrowing (arrows). Non-enhanced CT (middle row) demonstrates epidural hematoma (arrow). Bone reconstruction (lower row) reveals fracture crossing the transverse sinus groove (arrow).*

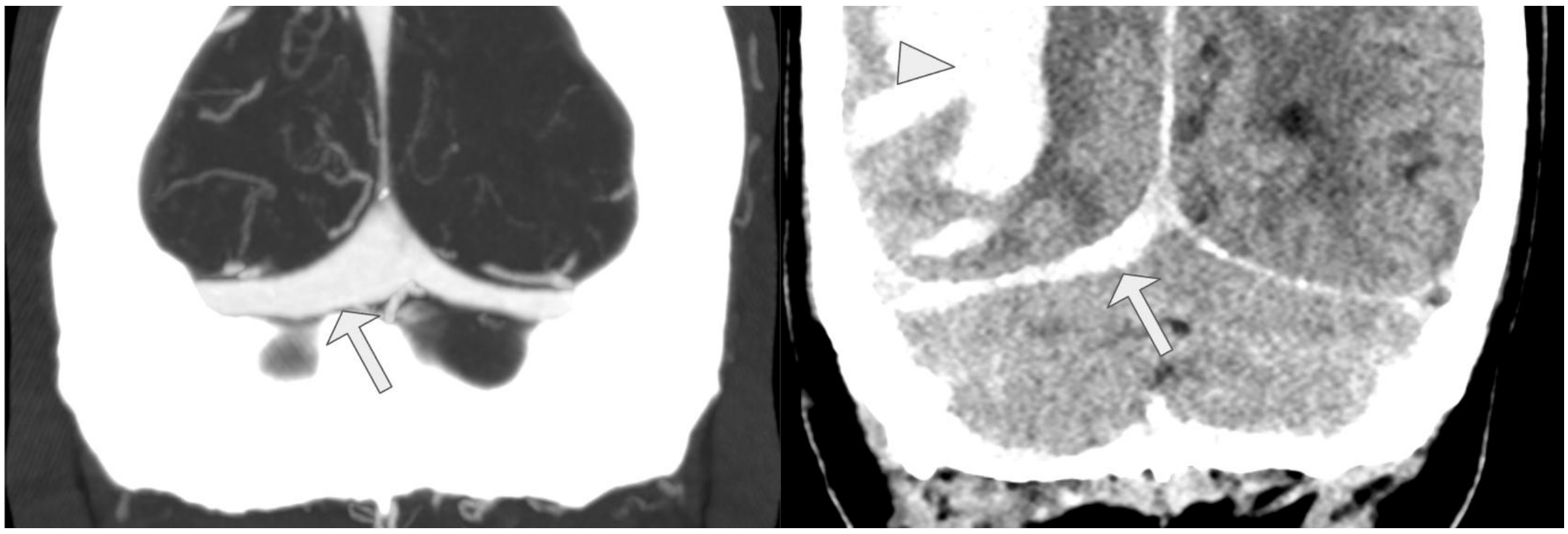

Supl Fig 9: *Tentorial subdural hematoma mimicking venous thrombosis. CT venography (a) shows patent sinuses (arrow). Non-enhanced CT (b) reveals thin tentorial subdural hematoma (arrow) and right occipital parenchymal hemorrhage (arrowhead), demonstrating the pitfall of misinterpreting subdural blood as sinus thrombosis.*

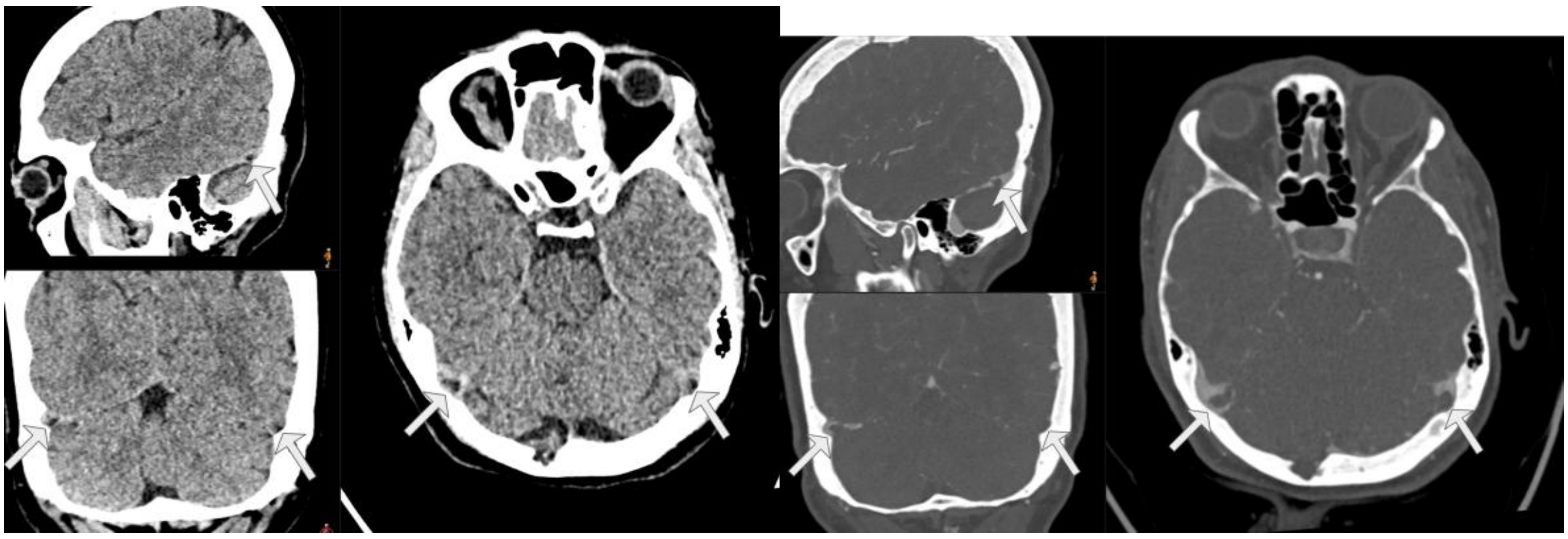

Supl Fig 10: *Arachnoid granulations as a common pitfall. CT venography (left) shows bilateral filling defects in transverse sinuses (arrows). Non-enhanced CT (right) demonstrates CSF attenuation within these structures (arrows), distinguishing arachnoid granulations from hyperattenuating thrombus.*

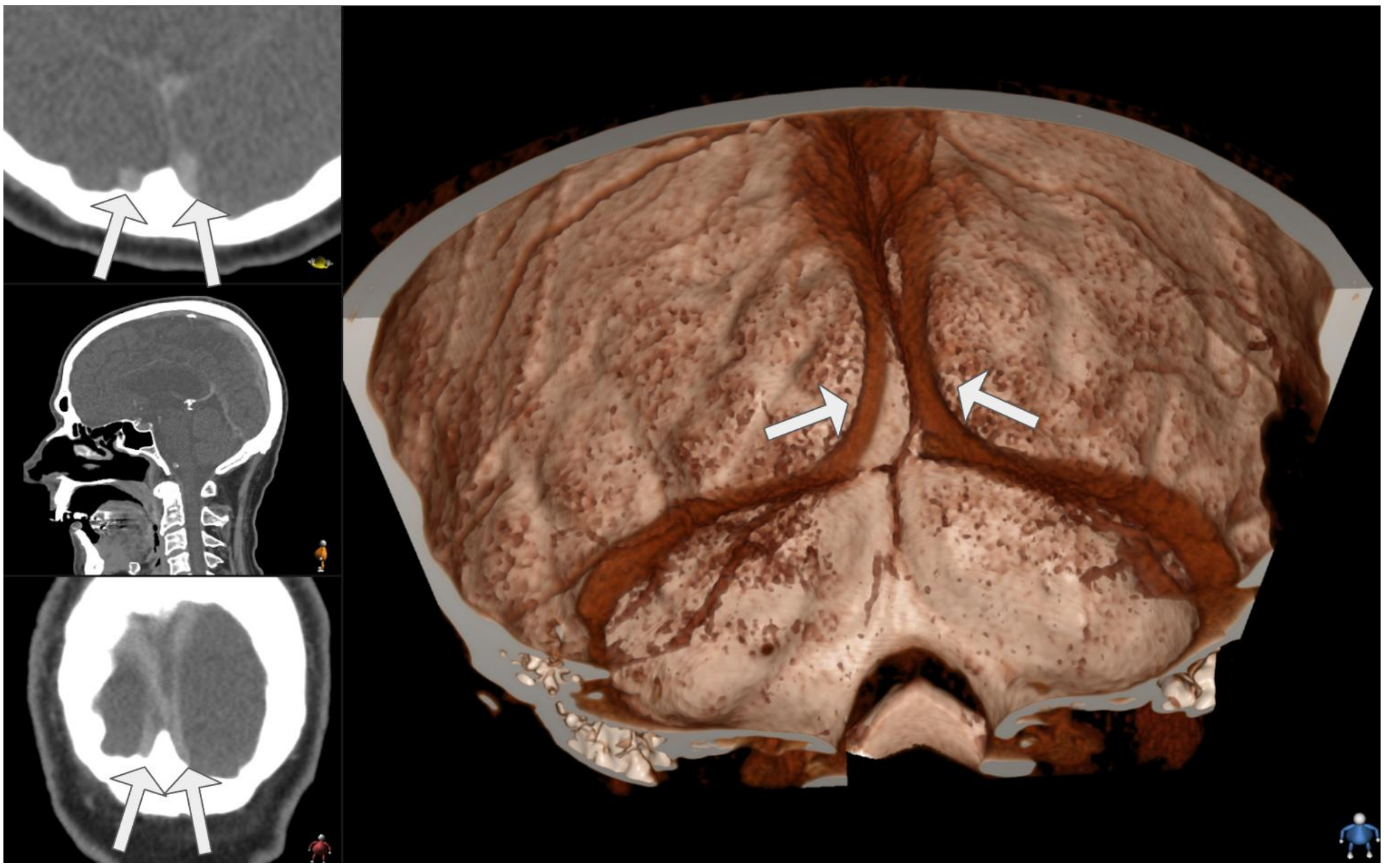

Supl Fig 11: *Split SSS variant. (a) Multiplanar CT venography reconstructions show duplicated SSS (arrows) lacking confluence at the torcula. (b) 3D volume rendering demonstrates the bifid configuration (arrows).*

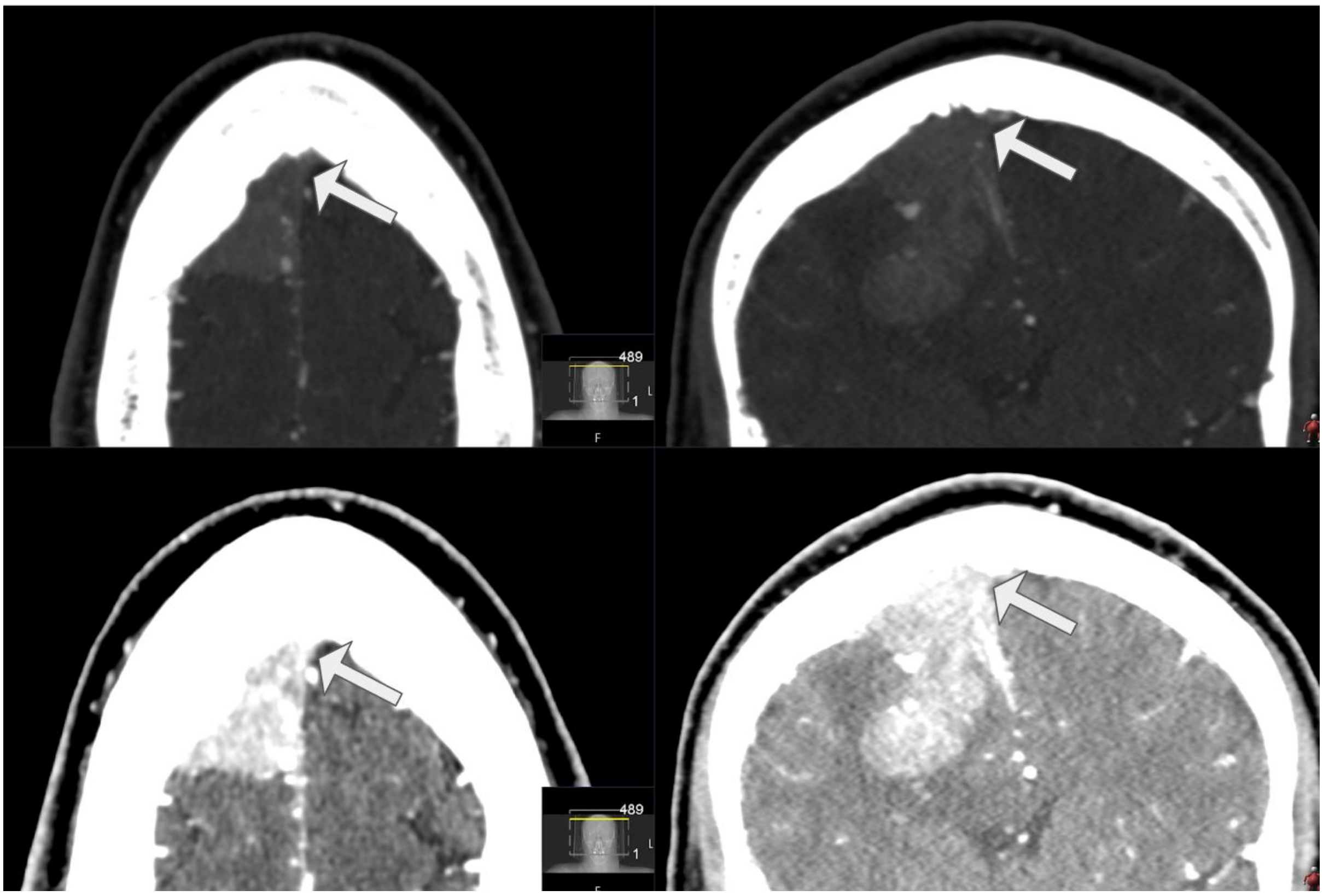

Supl Fig 12: *Parasagittal meningioma mimicking venous sinus thrombosis. Upper row: CT venography in (a) axial and (b) coronal planes demonstrates filling defect in the superior sagittal sinus (arrows). Lower row: (c) Axial and (d) coronal soft-tissue images show enhancing extra-axial mass (arrows) extending into the sinus lumen, confirming meningioma. Correlation between venographic and parenchymal images is essential to distinguish tumor invasion from thrombosis.*

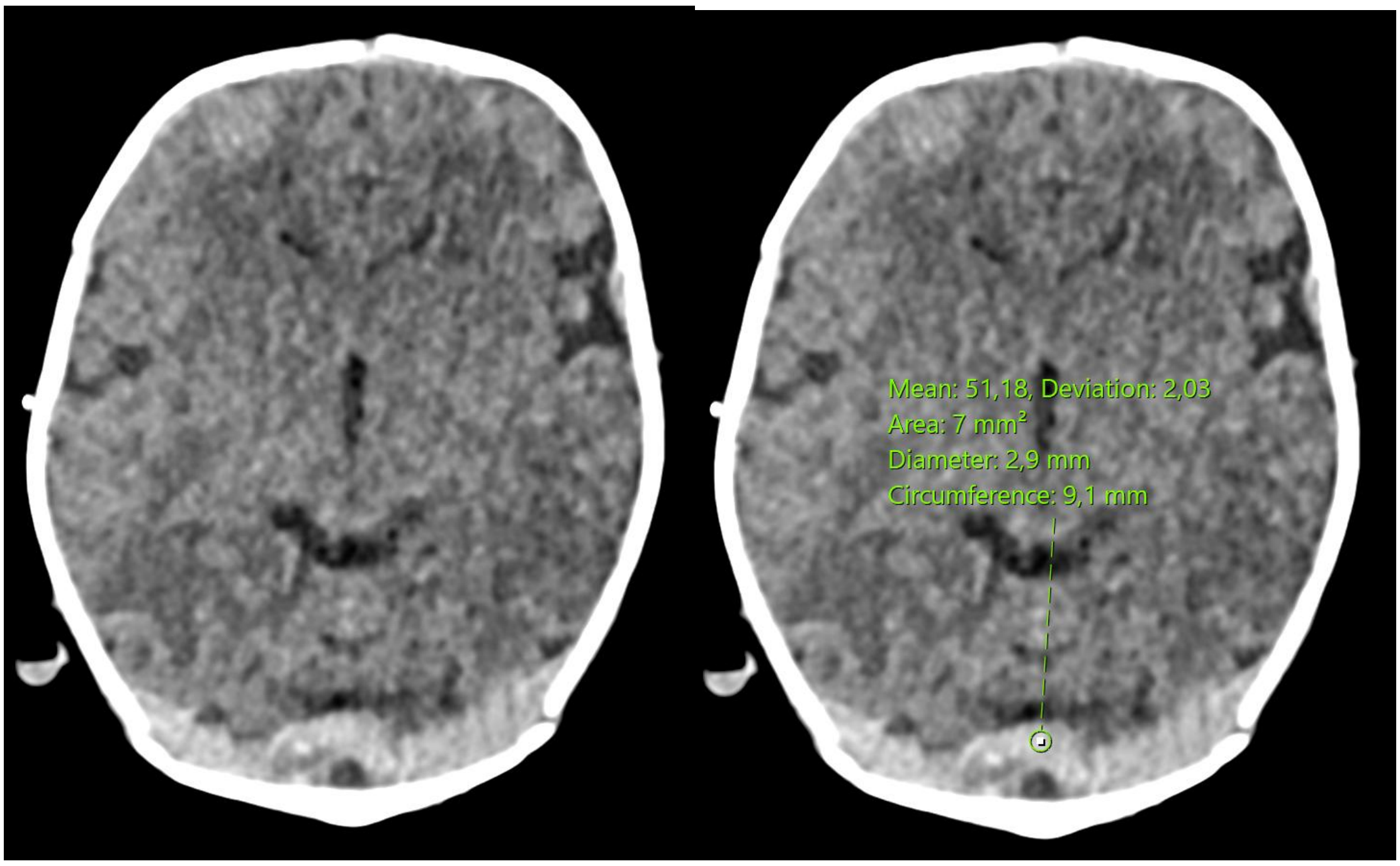

Supl Fig 13: *Normal neonatal dural sinus appearance mimicking thrombosis. (a) Non-enhanced CT in a newborn shows apparently hyperattenuating transverse sinuses (arrows) relative to the hypodense, immature brain parenchyma. (b) Hounsfield unit measurement within the sinus demonstrates mean attenuation of 51 HU (ROI shown), well below the threshold for acute thrombus (typically >58 HU), confirming patent sinuses without thrombosis.*

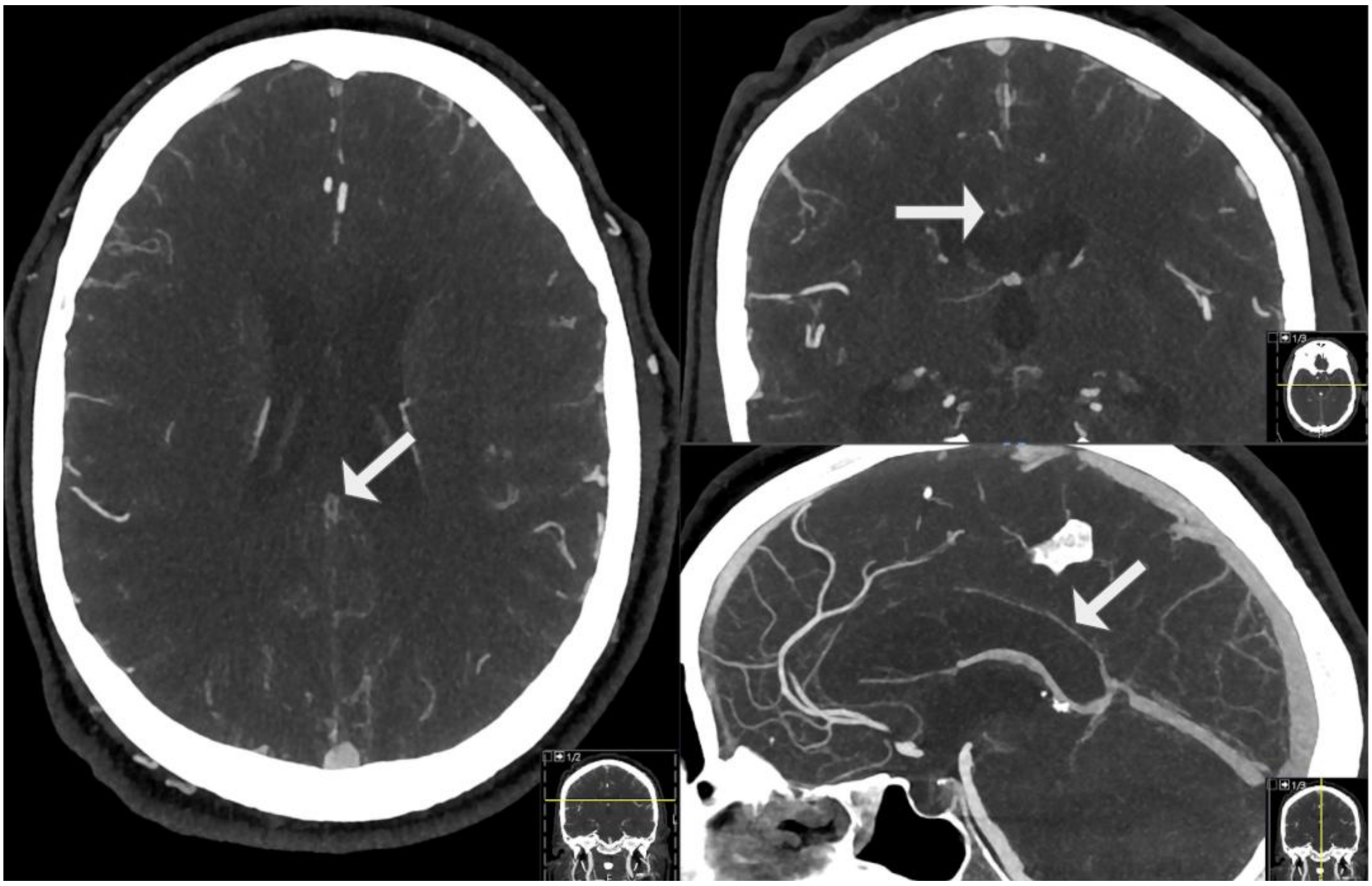

Supl Fig 14: *Hypoplastic inferior sagittal sinus. CT venography shows a thin, barely visible venous structure at the expected location of the inferior sagittal sinus (arrow), representing a normal anatomical variant.*

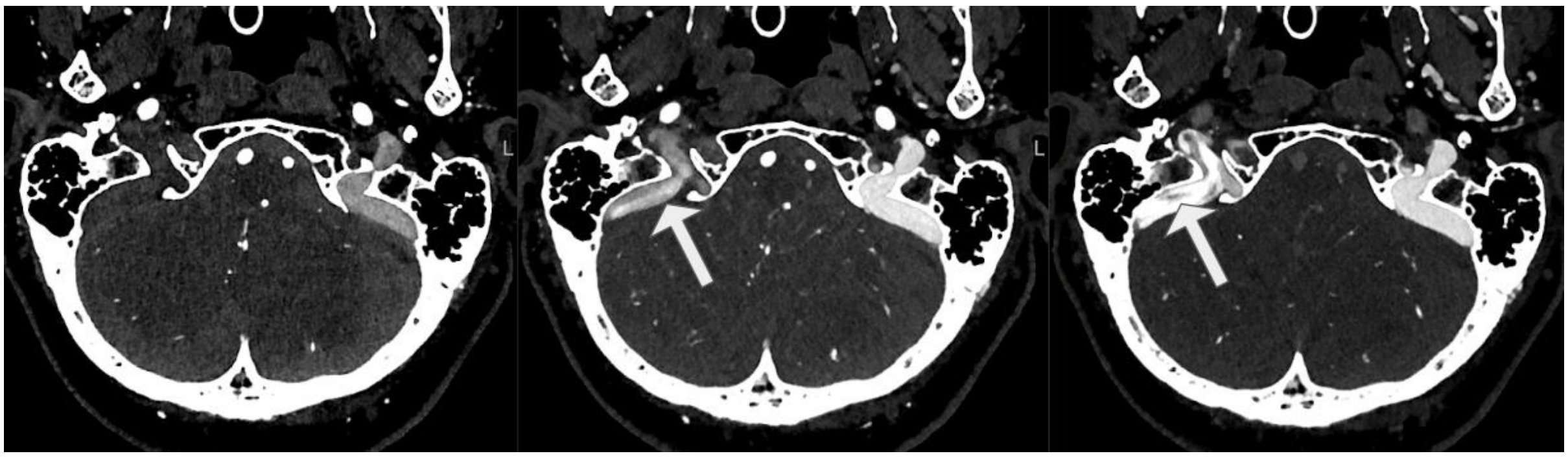

Supl Fig 15: *Contrast layering artifact in sigmoid sinus. Multiphase CT venography shows (a) arterial, (b) early venous, and (c) late venous phases with heterogeneous attenuation in right sigmoid sinus (arrows) from incomplete contrast mixing*

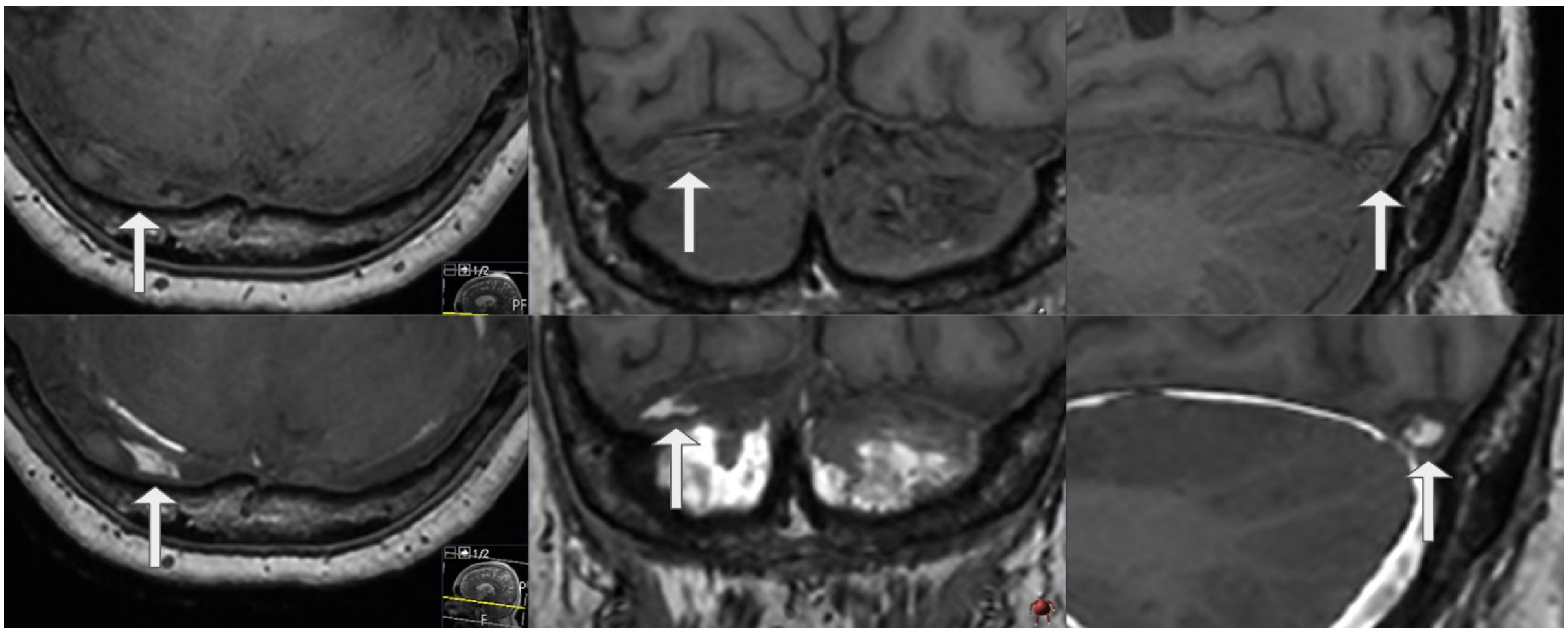

Supl Fig 16: *T1 signal evolution in venous thrombosis. Upper row (a-c): Day 0 shows isointense signal (arrows). Progressive T1 hyperintensity reflects methemoglobin formation in evolving thrombus. Lower row (d-f): Day 8 non-enhanced T1 shows hyperintense thrombus in right transverse sinus (arrows). The patient has concurrent subdural hematoma in the posterior fossa.*

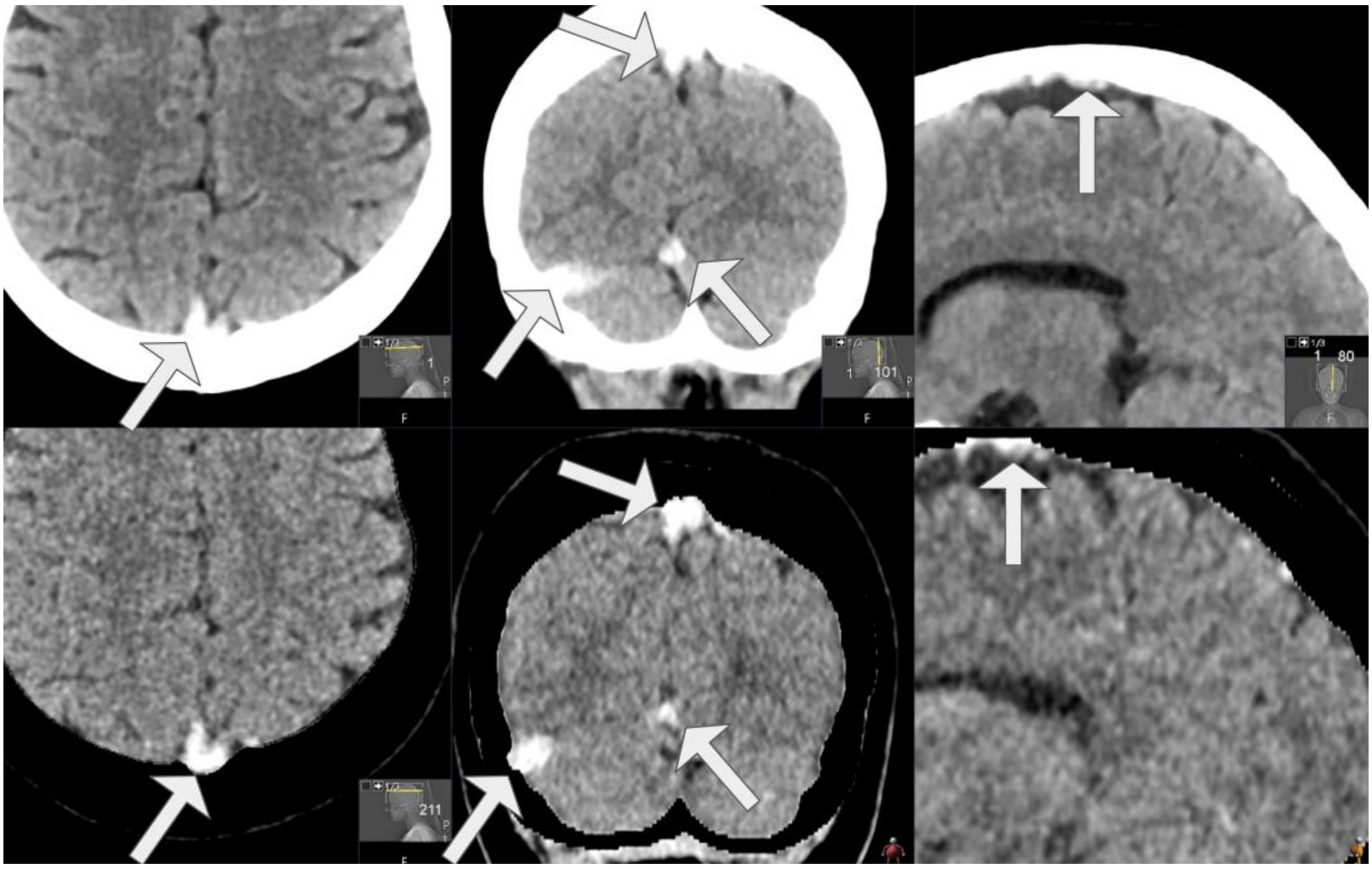

Supl Fig 17: *Bone removal for CVST visualization. Upper row (a-c): Standard non-enhanced CT (axial, coronal, sagittal) shows thrombus in superior sagittal and transverse sinuses (arrows). Lower row (d-f): Bone-subtracted images (same orientations) improve visualization of thrombosis extent (arrows).*

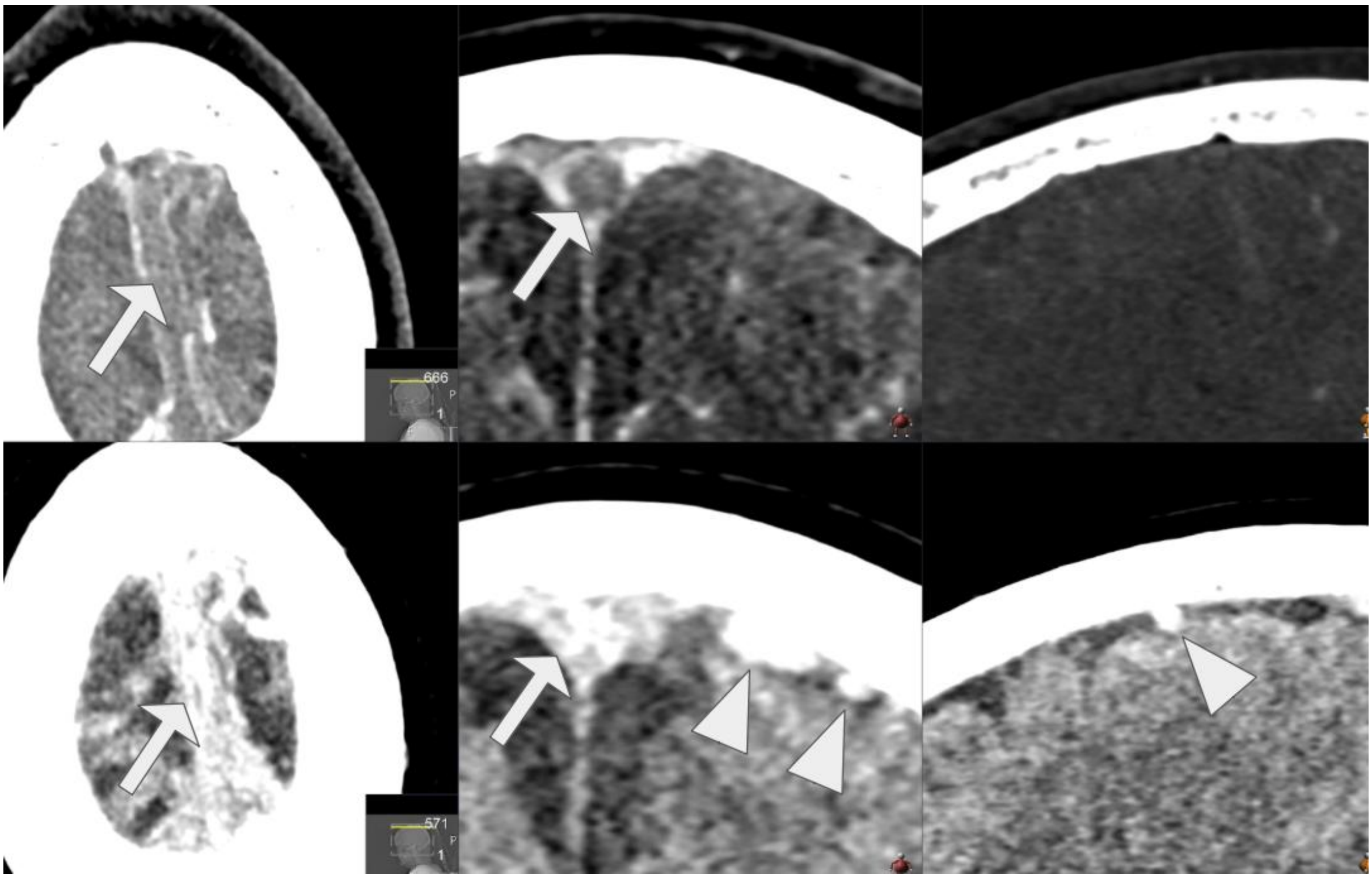

Supl Fig 18: *Superior sagittal sinus and left bridging vein thrombosis highlighting the value of non-contrast CT. Upper row: (a–c) CT venography (sagittal, axial, coronal) shows lack of contrast opacification in thrombosed segments (arrows), difficult to appreciate without correlation with non-contrast CT. Lower row: (d–f) Non-enhanced CT (sagittal, axial, coronal) clearly demonstrates hyperattenuating thrombus in the SSS (arrows) and left bridging vein (arrowheads). Note the elongated morphology of the bridging vein connecting cortical veins to the SSS. This case illustrates that when a thrombosed vessel shows complete lack of opacification on CTV without contrast surrounding the thrombus, non-contrast CT becomes essential to visualize the hyperattenuating thrombosed vessel itself and confirm the diagnosis.*

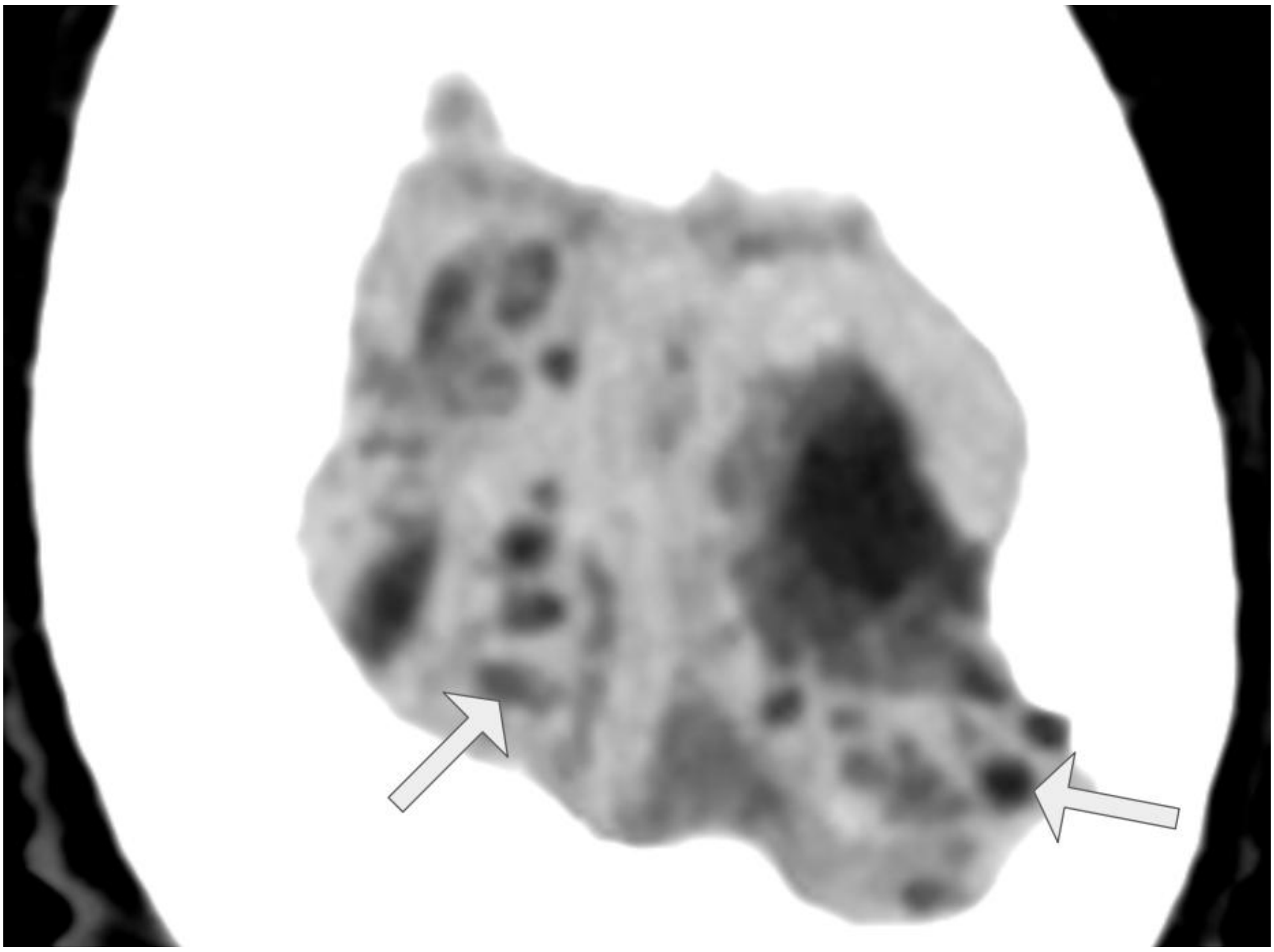

Supl Fig 19: *Normal appearance of parasagittal venous lakes filled with arachnoid granulations (arrows).*

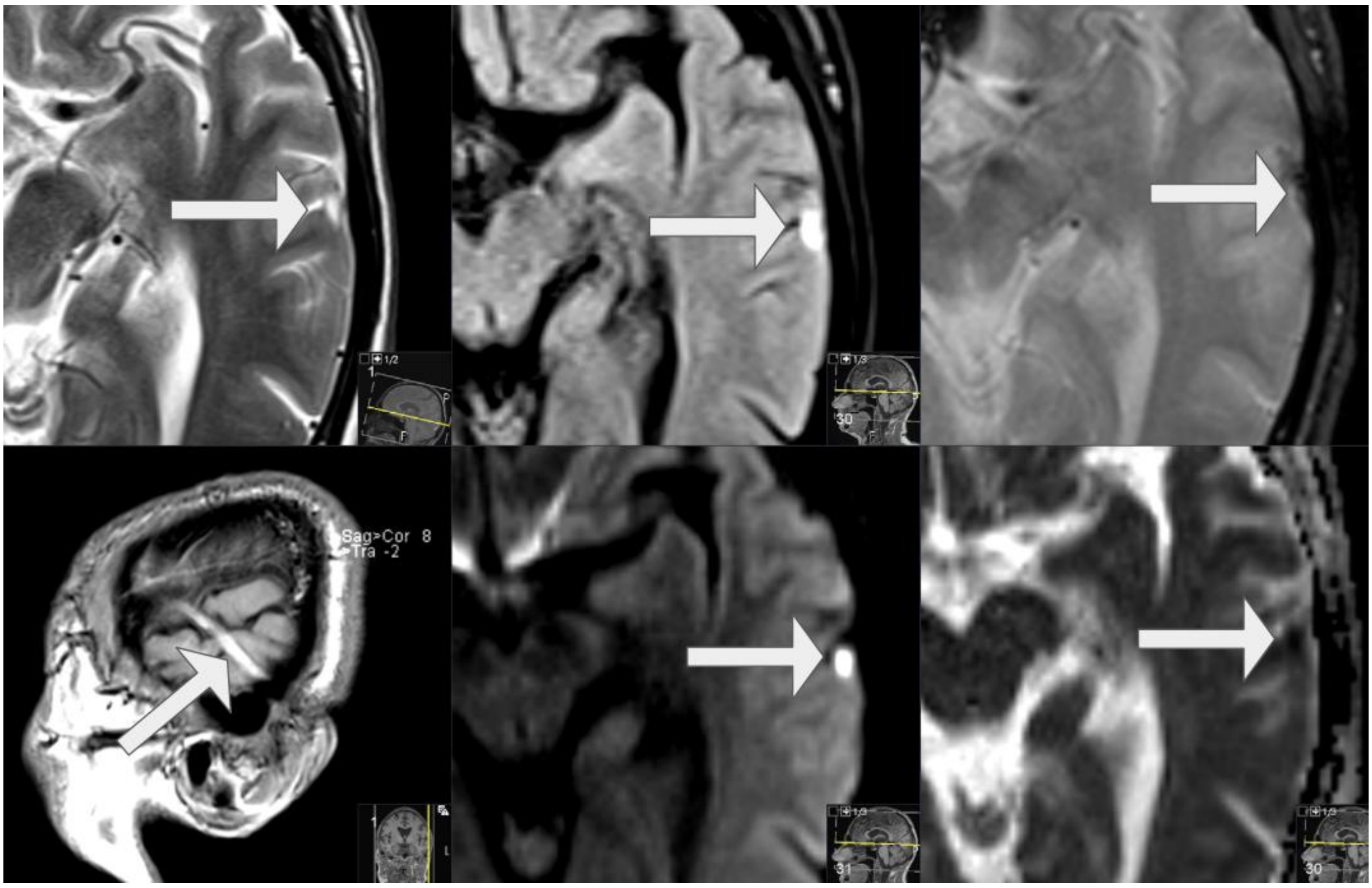

Supl Fig 20: *Vein of Labbé thrombosis. Upper row: (a) T2-weighted and (b) T2-FLAIR images show hyperintensity within the thrombus (arrows). (c) T2* demonstrates hypointensity within the thrombus (arrow). Lower row: (d) Sagittal T1-weighted image (without iv contrast) shows intrinsic hyperintensity within the thrombus (arrow). (e) DWI and (f) ADC map demonstrate restricted diffusion within the thrombus itself (arrows).*

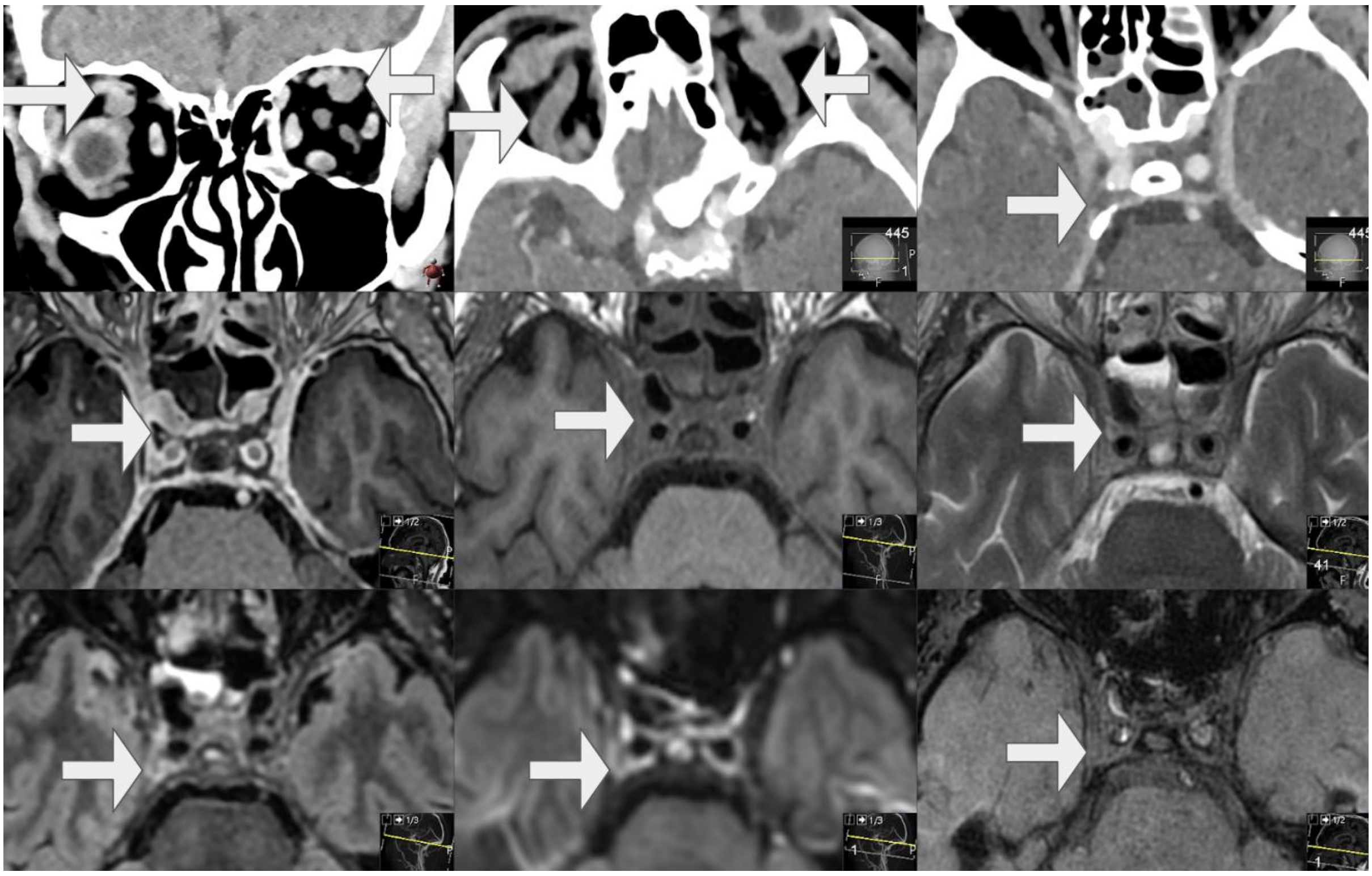

Supl Fig 21: *Septic cavernous sinus thrombosis from sinusitis. Upper row: (a,b) Contrast-enhanced CT shows dilated superior ophthalmic veins (arrows), (c) filling defect in cavernous sinus (arrow), widening of cavernous sinus. Middle row: (d) Post-contrast T1 shows lack of complete opacification, (e) pre-contrast T1 and (f) T2 show isointense sinus (arrows), (g) FLAIR heterogeneous hyperintensity (arrow). Lower row: (h) heterogeneous DWI hyperintensity (arrow), (i) SWI isointense (arrow). Atypical isointense T1/T2 signal indicates infectious material rather than typical thrombus.*

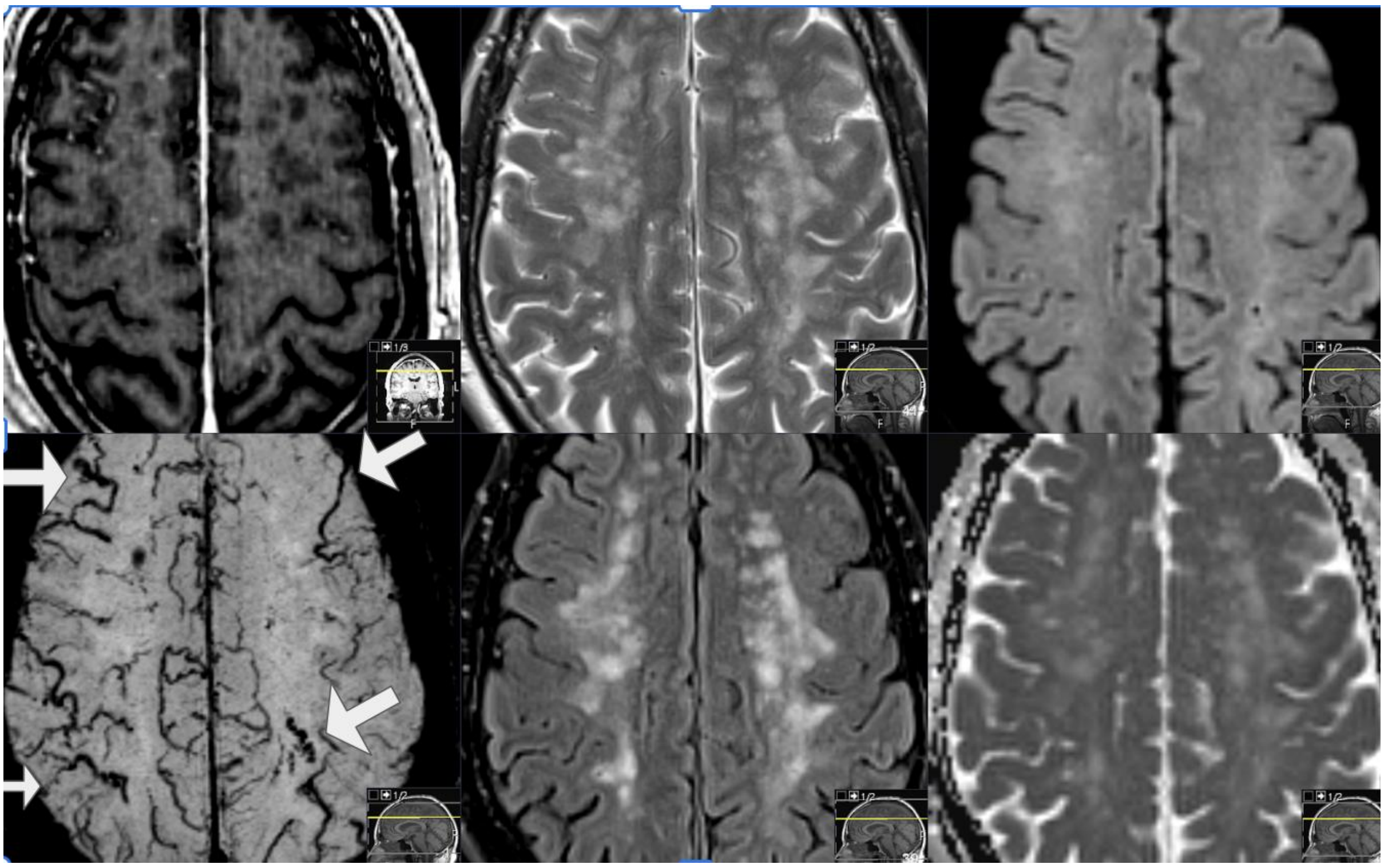

Supl Fig 22: *Dural arteriovenous fistulas mimicking venous stasis on SWI. Upper row: (a) T1 post-gadolinium, (b) T2, (c) DWI. Lower row: (d) SWI, (e) T2-FLAIR, (f) ADC. SWI shows prominent dilated cortical and medullary veins (arrows in d), mimicking the appearance of thrombotic venous stasis. However, other sequences demonstrate no evidence of thrombus or parenchymal complications. The venous engorgement results from arteriovenous shunting through multiple dural AV-fistulas rather than thrombotic occlusion.*

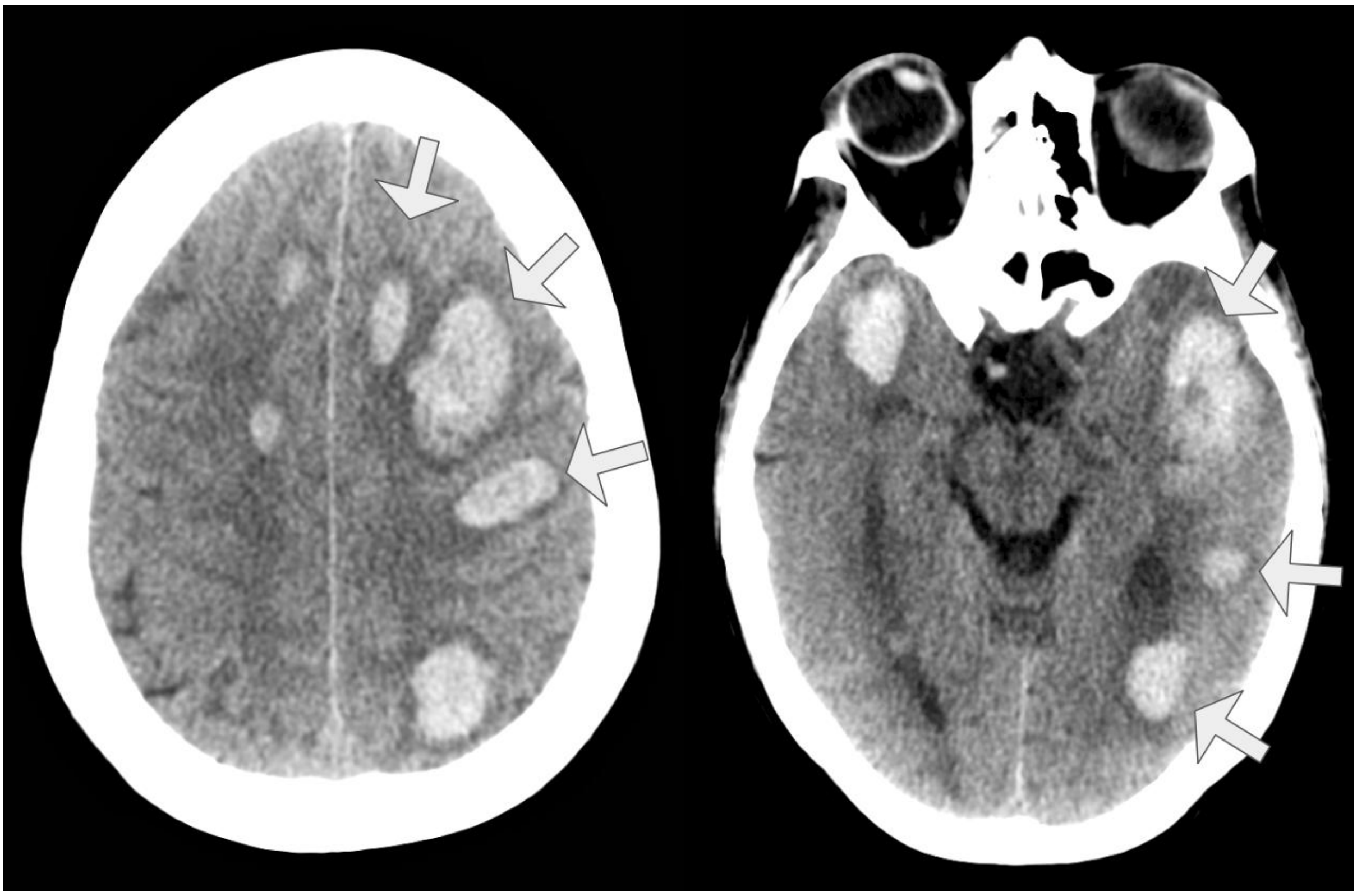

Supl Fig 23: *Characteristic centripetal hemorrhagic pattern of leukostasis. Non-enhanced axial CT images at (a) upper and (b) lower ventricular levels demonstrate multiple small hemorrhages oriented centripetally along medullary veins (arrows) in a patient with acute leukemia and hyperleukocytosis. This distinctive pattern results from leukostasis causing impaired blood flow through small medullary veins, leading to vessel rupture and hemorrhage tracking along venous drainage pathways from cortex toward deep gray matter and ventricles. High leukocyte count with concurrent thrombocytopenia predisposes to this complication. This pattern should prompt evaluation for underlying hematologic malignancy.*

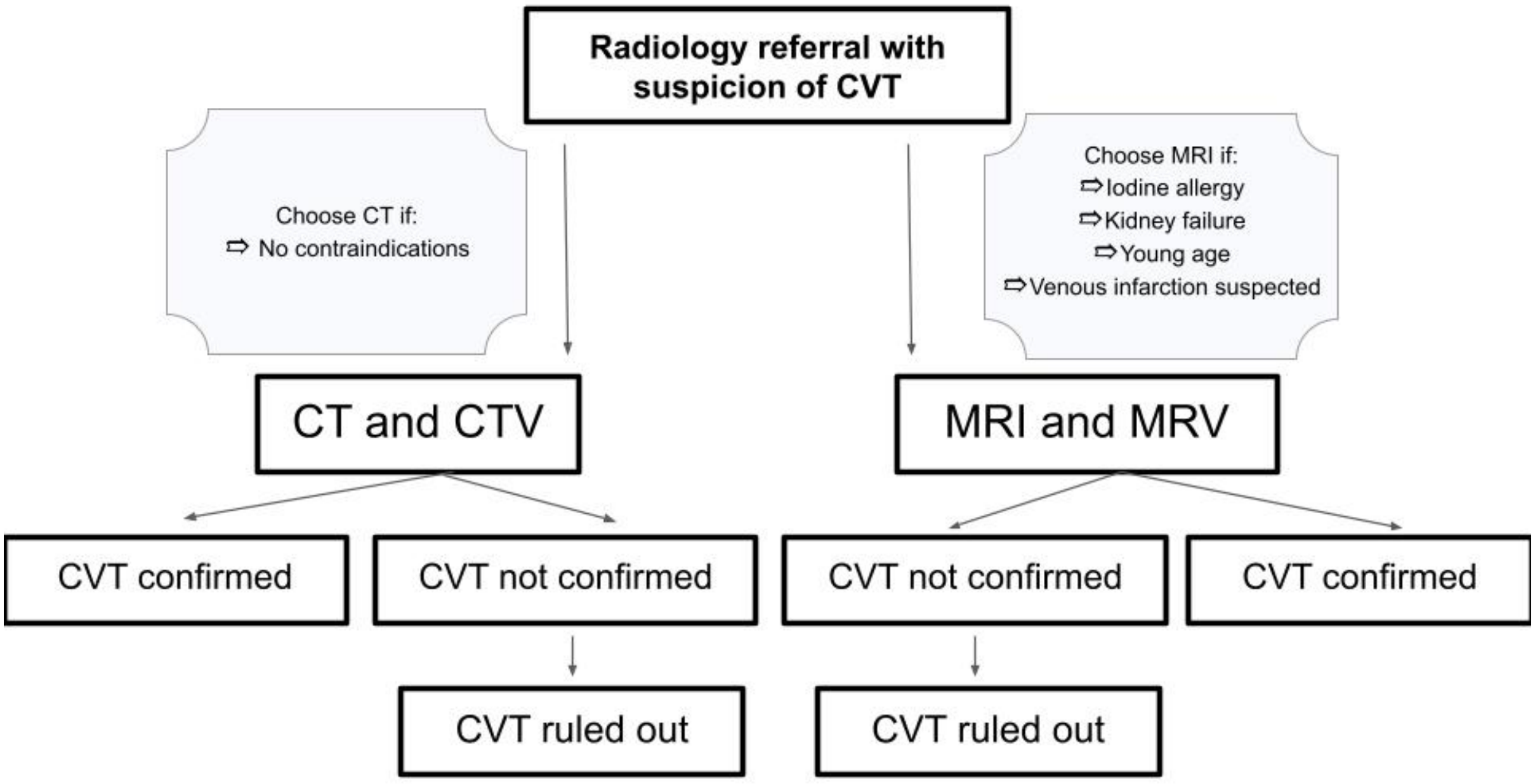

Supl Fig 24: *Figure 13. Decision flowchart for imaging in suspected cerebral venous thrombosis. Both CT with CTV and MRI with MRV reliably confirm or exclude CVT. MRI is preferred when specific clinical factors apply. CVT, cerebral venous thrombosis; CT, computed tomography; CTV, CT venography; MRI, magnetic resonance imaging; MRV, MR venography.*
